# Pediatric diabetes prediction using machine learning

**DOI:** 10.1038/s41598-025-24964-y

**Published:** 2026-01-15

**Authors:** Abeer El-Sayyid El-Bashbishy, Hazem M. El-Bakry

**Affiliations:** 1 Information Systems Department, Faculty of Computer and Information Sciences, Mansoura University, Mansoura, Egypt; 2 Professor of Information Systems Department, Faculty of Computer and Information Sciences, Mansoura University, Mansoura, Egypt

**Keywords:** Medical research, Engineering

## Abstract

Diabetes is a chronic condition that affects a substantial portion of the global population and is linked to elevated mortality rates and a range of severe health complications. Despite its clinical importance, progress in diabetes research is often constrained by the limited availability of comprehensive datasets and robust predictive models. To address these challenges, researchers are increasingly turning to big data analytics and machine learning (ML) methodologies. This study presents the development of an ML-based system aimed at predicting the likelihood of diabetes and classifying its various types. A novel dataset, termed Diabetes Types Dataset, was constructed by integrating four heterogeneous dataset sources: paediatrics data from the Mansoura University Children Hospital repository, the Pima Indian Diabetes (PIMA) dataset, the Pone dataset, and a Gestational Diabetes dataset. The classification of diabetes types was approached as a multiclass problem using a suite of supervised ML algorithms, including Artificial Neural Networks (ANN), Logistic Regression, Naive Bayes, Decision Trees, Adaptive Boosting, Random Forests, Gradient Boosting, Support Vector Machines, and K-Nearest Neighbors. Model performance was evaluated using several metrics: Accuracy, Precision, Mean Squared Error, and Area Under the Receiver Operating Characteristic Curve. Among the models tested, the ANN classifier demonstrated the highest accuracy, achieving a peak performance of 99.98%. Further validation was conducted using an external dataset referred to as diabetes_prediction, which confirmed the model’s robustness with consistent accuracy. Additionally, the proposed system was applied to a publicly available dataset, diabetes_Dataset, containing 34 features used to predict 12 distinct types of diabetes efficiently. The results suggest that this ML-driven approach can significantly enhance the ability of healthcare professionals to detect and classify diabetes types, thereby supporting early intervention and improved disease management.

**Supplementary Information:**

The online version contains supplementary material available at 10.1038/s41598-025-24964-y.

## Introduction

**Fig. 1:**
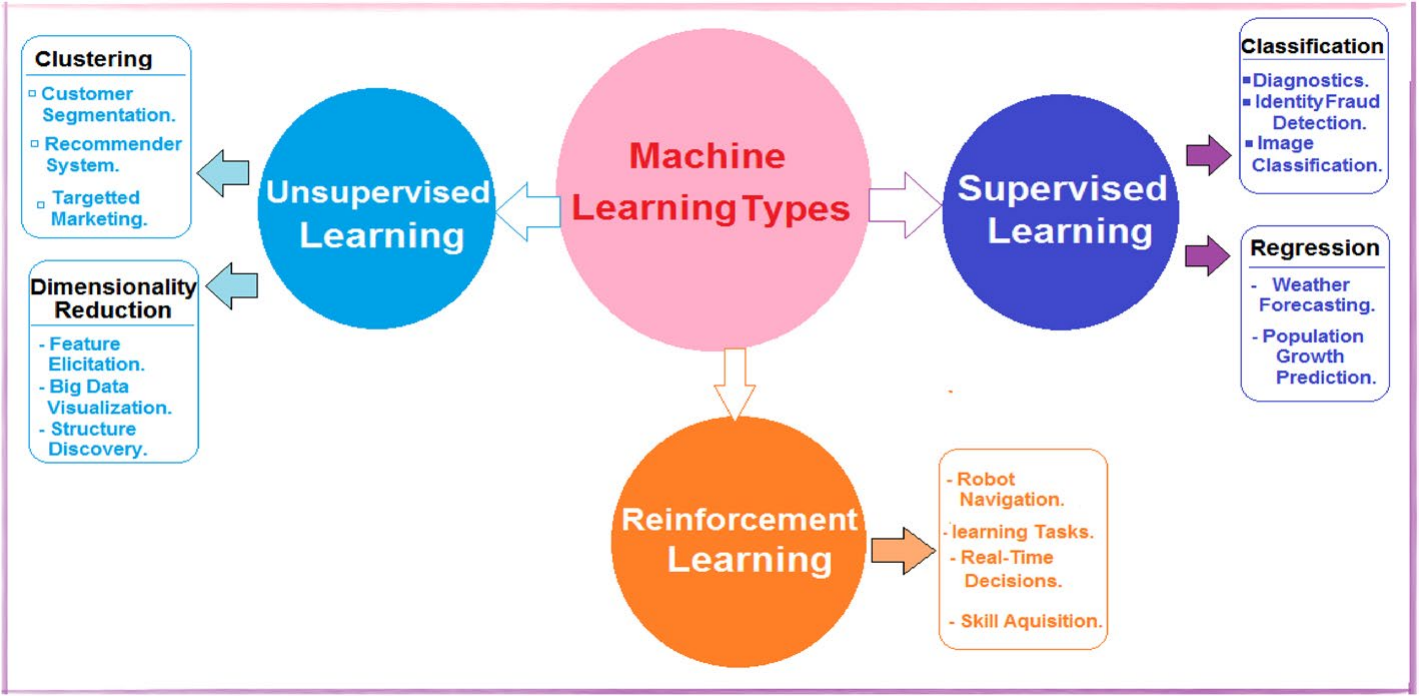
Machine learning types.

The World Health Organization has reported that approximately 1.6 million deaths occur annually due to diabetes^1^. Diabetes, characterized by insufficient insulin, is a complex condition affecting various organs such as the liver, muscles, and adipose tissue, as insulin regulates glucose production^2^. Inadequate insulin levels result in elevated blood glucose, leading to potential organ damage, including the eyes, cardiovascular system, and nerves^3^. Diabetes manifests as various types, primarily Type 1, Type 2, and Gestational^4^. Type 2 diabetes often stems from obesity-induced insulin resistance, while type 1 diabetes arises from immune system attacks on pancreatic beta cells^5^. Both types of hyperglycemia are due to insulin deficiency or resistance^6^. Type 1 diabetes predominantly affects children and young adults, whereas type 2 diabetes is prevalent in adults^7^. Gestational diabetes, diagnosed during pregnancy, poses risks to both maternal and fetal health but can be mitigated with early detection and management^8^. Monitoring blood glucose levels and adhering to medical advice are crucial for diabetic individuals^9^. The typical blood glucose level can vary based on factors such as age, family medical history, individual health status, and dietary habits. Glucose, derived from food, serves as the primary energy source for the body. Various laboratory examinations, including tests for HbA1c, C-peptide, glucose, insulin, acetone, and blood gases, are necessary for diabetes detection. A positive result in the urine indicated the presence of acetone. Hemoglobin A1c (HbA1c) serves as a diagnostic tool, reflecting the average glucose level over the past two to three months^10^. The C-peptide test aids in identifying pancreatic cell destruction by the immune system, particularly in pediatric diabetes patients. Abnormal C-peptide levels may indicate poor insulin utilization by the body. Blood gas analysis accurately measures oxygen and carbon dioxide levels within the body. Diabetes manifests as several chronic symptoms such as excessive hunger, thirst, frequent urination, neuropathy, nephropathy, retinopathy, cardiovascular issues, peripheral vascular disease, dental complications, stroke, diabetic foot syndrome, encephalopathy, hyperthyroidism, adrenal gland tumors, cirrhosis of the liver, glucagonoma, persistent vomiting, severe abdominal pain, metabolic acidosis, fatigue, dysuria, diabetic ketoacidosis, weight loss, and various other health complications. Early detection facilitates effective diabetes management, involving a balanced fitness regimen, proper treatment, and nutritious eating habits^11^. Preventive measures encompass addressing risk factors and fostering healthy lifestyles, including regular physical activity, limited intake of processed foods and refined carbohydrates, adequate protein consumption, and ample fruits and vegetables. Risk factors include maternal glucose levels during pregnancy and childhood obesity. Type 1 diabetes treatment typically involves insulin administration via injections or insulin pumps, frequent blood glucose monitoring, and carbohydrate counting. Additionally, hyperbaric oxygen therapy has recently gained attention for its ability to enhance healing processes by elevating oxygen levels in the body. Pressurized oxygen serves as a complementary therapeutic approach for diabetic foot care, facilitating increased blood flow and oxygen delivery to damaged cells and tissues, thereby aiding in their restoration and effective recovery. Recently, the proliferation of medical knowledge has surged alongside advancements in artificial intelligence methodologies, which are adept at managing and processing this wealth of information to effectively enhance diabetes management practices. The fundamental objective of intelligent learning from accumulated data is to enable computers to autonomously acquire knowledge without human intervention. ML has emerged as a computational technique that harnesses experiences, historical data, and repository information to cultivate intelligence^12^. ML proves invaluable in diagnosing diabetes; it boasts various types of learning, such as supervised learning, unsupervised learning, and reinforcement learning, as depicted in Fig. 1. Supervised learning algorithms are trained using examples, incorporating both input and target data into the training dataset. Classification and regression exemplify supervised learning techniques. Conversely, unsupervised learning algorithms discern latent patterns within training data without relying on labeled information to analyze and extract significant features. Clustering and dimensionality reduction represent unsupervised learning methodologies. Reinforcement learning empowers agent systems to learn within interactive environments, guided by feedback mechanisms employing reward and penalty policies. Rewards, whether positive or negative, shape the behavior of the agent or system, with applications ranging from robot navigation to real-time decision-making^13^.

The remainder of this paper is organized as follows: in the second section, we present the related work. Section Methods details the proposed architecture and the corresponding features through a comparison of nine classification techniques. The results are presented in Section Train phase, and the conclusions are presented in Sect.on Machine learning multi-class classification.

## Related work

This study introduces two ensemble models based on stacking techniques, one employing traditional ML algorithms and the other utilizing a deep neural network (DNN) for the early detection of diabetes^14^. The models are developed by integrating three data sources: the Pima Indian Diabetes (PID) dataset, a simulated dataset, and a local healthcare dataset. By combining the predictions of multiple base classifiers, the ensemble models aim to improve predictive accuracy and robustness. Performance evaluation was conducted using both a train-test split and 5 fold cross-validation. The DNN-based ensemble demonstrated the highest performance on the simulated dataset, achieving an accuracy of 95.5%, precision of 94%, recall of 97%, and an F1-score of 96%. On the PID dataset, the stacked ML models achieved an accuracy of 75.03% with the train-test split and 77.10% with cross-validation. Overall, the proposed approach achieved accuracy levels between 92 and 95%, with corresponding precision, recall, and F1-scores ranging from 88 to 96%, highlighting its potential for reliable early-stage diabetes detection.s

This study seeks to improve the generalizability and predictive accuracy of diabetes classification models by addressing the limitations inherent in single-population datasets^15^. To this end, the researchers integrated two publicly available datasets: the Pima Indian Diabetes (PID) dataset comprising 768 samples, and the German Society dataset containing 2000 samples. Ensemble learning methods, specifically RF and GB, were employed to evaluate model performance on both individual and combined datasets. The findings indicated that models trained on the merged dataset outperformed those trained on individual datasets, with GB and RF achieving accuracies of 0.991 and 0.988, respectively, compared to RF of 0.817 on PID and GB of 0.996 on the German dataset.

This study proposes a pipeline-based multi-classification framework for predicting diabetes status categorized as diabetic, non-diabetic, and prediabetic using an imbalanced dataset from Iraqi patients^16^. To overcome challenges such as limited labelled data, missing values, and class imbalance, the framework incorporates extensive preprocessing steps, including duplicate removal, imputation, normalization, standardization, feature selection, and k-fold cross-validation. Several ML algorithms KNN, SVM, DT, RF, AB, and NB are applied, with a weighted ensemble model guided by AUC introduced to enhance predictive performance. Hyperparameter optimization is conducted via grid search and Bayesian methods. The proposed ensemble model outperforms individual classifiers, achieving an accuracy of 0.9887, a precision of 0.9861, a recall of 0.9792, an F1-score of 0.9851, and an AUC of 0.999. These findings indicate the framework’s robustness and scalability, with strong potential for broader application in diabetes prediction across diverse populations.

This study presents an automated diabetes prediction system leveraging ML techniques on a combined dataset comprising the PID dataset and additional records from 203 female patients in Bangladesh^17^. Mutual information is utilized for feature selection, and a semi-supervised learning approach employing Extreme Gradient Boosting (XGBoost) is adopted to estimate insulin levels. To mitigate class imbalance, oversampling techniques such as SMOTE and ADASYN are applied. A range of classifiers, including DT, SVM, RF, LR, KNN, and several ensemble models, are evaluated. The XGBoost model combined with ADASYN achieves the highest performance, with 81% accuracy, an F1-score of 0.81, and an AUC of 0.84. To assess generalizability, domain adaptation techniques are employed, while model interpretability is addressed using explainable tools such as SHAP and LIME. The system is further integrated into both a web-based interface and an Android application, enabling real-time diabetes prediction and enhancing its applicability in clinical and remote healthcare settings.

This study focuses on early diabetes prediction through the application of nature-inspired metaheuristic algorithms and includes both case studies and a comprehensive review of existing predictive models^18^. Various bio-inspired optimization techniques, such as Ant Colony Optimization, Bat Algorithm, Cuttlefish Algorithm, Elephant Herd Optimization, and Artificial Bee Colony, are employed to enhance classifier performance via hyperparameter tuning. A hybrid Bat Algorithm is specifically used to optimize several classification models, while SMOTE is applied to address class imbalance. Among the evaluated models, a voting classifier enhanced with both SMOTE and the Bat Algorithm achieved the highest accuracy of 98%. The findings highlight the effectiveness of nature-inspired optimization strategies in improving prediction performance and further explore the integration of dietary recommendations based on predictive outcomes, suggesting a promising direction for personalized diabetes management.

This study emphasizes the early prediction of diabetes using both statistical and non-statistical ML techniques, applied to the PID Dataset comprising 768 patient records^19^. Key predictive features include age, BMI, and blood glucose levels, which are recognized as critical risk indicators. A diverse set of classification algorithms, including LR, DT, RF, KNN, NB, SVM, GB, and ANN, is evaluated for its predictive effectiveness. Among these, the ANN model demonstrated the highest accuracy at 78.57%, followed by RF at 76.30%. The results highlight the potential of ML as a robust, data-driven approach for early diabetes risk assessment, enabling timely medical intervention and improved disease management.

This study introduces an advanced diabetes prediction framework that integrates conventional ML models, including LR, SVM, NB, and RF, with ensemble methods such as AB, GB, Extra Trees, and XGBoost^20^. Central to the framework is DNet, a novel hybrid deep learning architecture that combines Convolutional Neural Networks (CNN) for feature extraction with Long Short-Term Memory (LSTM) layers to capture temporal dependencies. DNet incorporates convolutional and residual blocks with skip connections, as well as Batch Normalization and Dropout for improved regularization and generalization. The framework is evaluated on a real-world Kaggle diabetes dataset using cross-validation and performance metrics such as precision, recall, F1-score, and ROC-AUC. DNet significantly outperforms all baseline models, achieving an accuracy of 99.79% and an AUC-ROC of 99.98%. These results underscore the model’s robustness and its strong potential for real-world application in early and accurate diabetes diagnosis.

ANN was utilized for diabetes prediction, with model training, validation, and testing conducted using the Just Neural Network software environment^21^. The dataset consisted of 1004 records with 9 input features, collected from the Association of Diabetic City of Urmia. Training was performed using the backpropagation algorithm to reduce prediction error. The model achieved an average error rate of 0.01 and a prediction accuracy of 85.09%, demonstrating its effectiveness in identifying diabetic cases.

The dataset attributes, including age, gender, family history, BMI, and blood glucose levels, exhibit interrelated patterns relevant to diabetes prediction^22^. ML algorithms were applied for the detection and classification of diabetes, with further potential for enhancing predictive accuracy through the identification of diabetes type and the estimation of comorbid disease risk. Two datasets, the PIMA dataset and a Clinical Survey (CS) dataset, were utilized in the study. Diabetes classification into pre-diabetes and diabetes categories was performed using multiple classification algorithms. Preprocessing methods such as data augmentation and sampling were employed to improve model performance. Among the tested algorithms, including RF, Light Gradient Boosting Machine (LGBM), GB, SVM, DT, and XGBoost, the LGBM classifier achieved the highest accuracy of 95.20%.

Researchers employed various machine learning models including ANN, KNN, NB, AB, LR, RF based on DT, and SVM to predict diabetes^23^. Using Pearson’s correlation for feature analysis and the WEKA tool for data processing, missing values in the PID dataset were imputed with mean values. The study achieved an accuracy of 83% with a low error rate.

Using the PID dataset, researchers applied ANN, data mining, and ML algorithms to predict diabetes^24^. Missing values were imputed with mean values, LR and SVM models achieved accuracies of 78.86% and 78.29%, respectively. The ANN model, configured with multiple epochs and two hidden layers utilizing the ReLU activation function, demonstrated superior performance with an accuracy of 90.1%.

Building on prior research, it is noted that all patients in the widely utilized PIMA dataset are female, have experienced pregnancies, and are aged 21 years or older. Due to the prevalence of missing values in this dataset, thorough preprocessing is essential to prevent inaccurate predictions. This study focuses on the early detection of pediatric diabetes and aims to identify the key factors affecting the health of a younger patient population. Accordingly, the researchers seek to develop an optimized machine learning algorithm designed to predict diabetes with improved accuracy.

## Methods

**Fig. 2:**
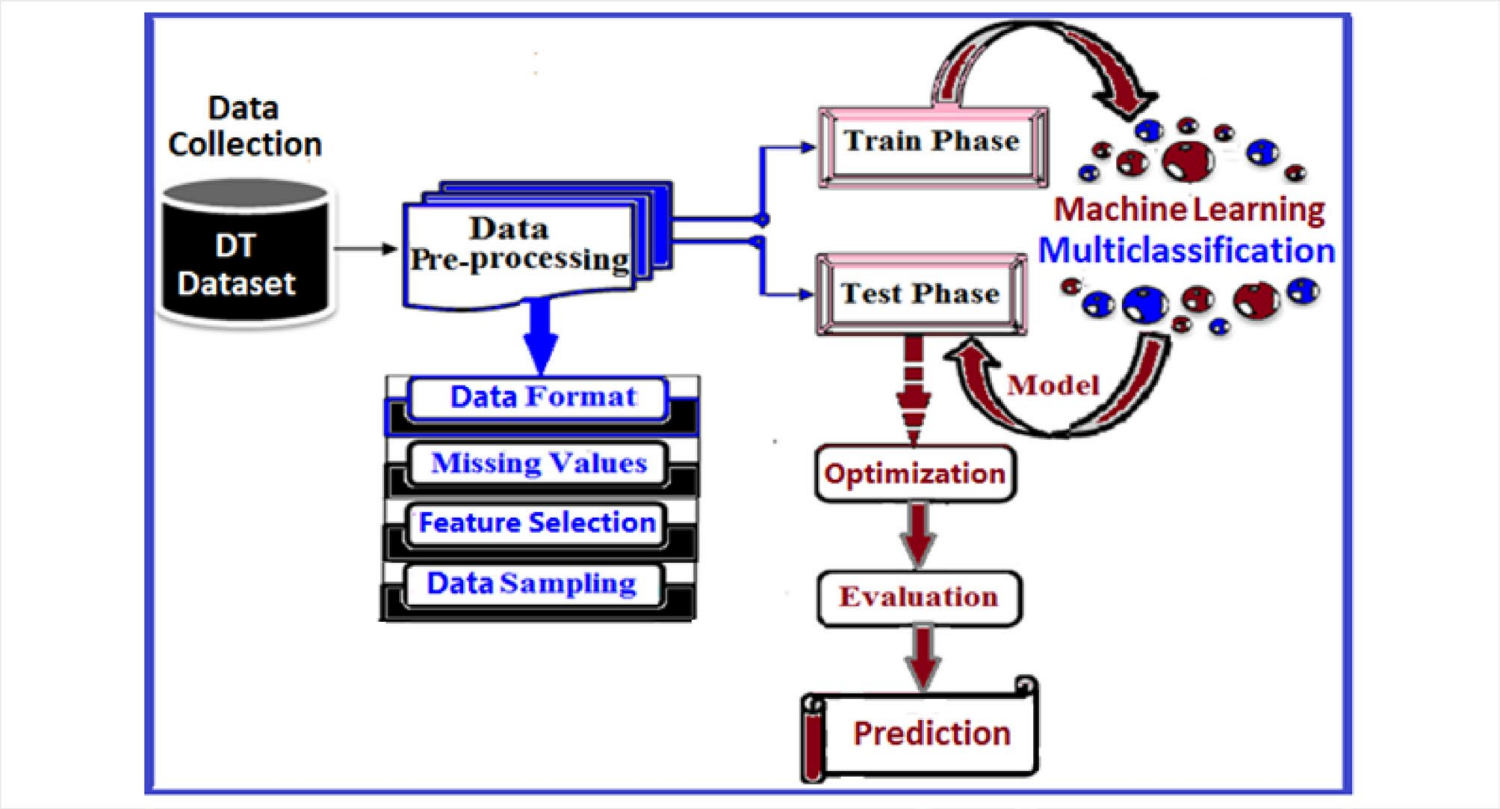
The diabetes data types system architecture.

This study introduces a novel model for multiclassification of diabetes types. The model encompasses a series of steps, beginning with data collection and proceeding through preprocessing, and data analysis-based feature extraction followed by a training/testing phase. The next phase of the ML multiclassification process is followed by evaluating the classifier’s performance, optimization, and prediction phases. The system architecture is graphically presented in Fig. 2.

### Data collection

Data forms the foundation of ML models, shaping their effectiveness. Gathering the appropriate data, both in terms of quality and quantity, is pivotal for constructing an optimal model. The reliability of the collected data plays a crucial role across all phases of model classification, ultimately influencing the quality of predictions. This phase involved selecting significant data features and determining the necessary sample size, guided by assumptions regarding the most relevant attributes to diabetes. Therefore, the quality of data directly impacts the performance of the model, contributing to its overall efficacy. We declare the detailed explanation of how to unify different datasets into the new DTD dataset as shown in online supplementary material.

### Dataset

The proposed system employs the newly developed DTD dataset, which integrates data from four distinct sources: the Pediatrics, PID, Pone, and Gestational diabetes datasets, resulting in a comprehensive dataset of 5312 patients with 13 attributes including Age, Sex, BPressure, NPregnancies, BMI, HbA1c, Insulin, POGTT, FOGTT, PGlucose, FGlucose, Diagnosis and DiagnosisType. The Pediatrics dataset, sourced from Mansoura University Children’s Hospital in Egypt, includes 619 patients aged 1 to 19. The PID dataset, obtained from the UCI Repository, contributes data from 768 patients. The Pone dataset, collected from hospitals in Thanjavur district, Tamil Nadu, India, includes 400 patients with 23 features. The Gestational dataset comprises data from 3012 patients with 17 attributes, gathered by diabetes researchers. The goal of the DTD dataset is to identify key factors influencing diabetes occurrence within a multiclass classification framework. We use two external datasets: the first external dataset is diabetes_prediction_dataset, containing 100,000 patient records and 9 features, namely gender, age, hypertension, heart_disease, smoking_history, bmi, HbA1c_level, blood_glucose_level, and diabetes which was also utilized, comprising 91,501 non-diabetic and 8499 diabetic cases. The second external dataset is diabetes_Dataset, containing 34 features namely: Target, Genetic Markers, Autoantibodies, Family History, Environmental Factors, Insulin Levels, Age, BMI, Physical Activity, Dietary Habits, Blood Pressure, Cholesterol Levels, Waist Circumference, Blood Glucose Levels, Ethnicity, Socioeco2mic Factors, Smoking Status, Alcohol Consumption, Glucose Tolerance Test, History of PCOS, Previous Gestational Diabetes, Pregnancy History, Weight Gain During Pregnancy, Pancreatic Health, Pulmonary Function, Cystic Fibrosis Diagnosis, Steroid Use History, Genetic Testing, Neurological Assessments, Liver Function Tests, Digestive Enzyme Levels, Urine Test, Birth Weight, and Early Onset Symptoms used to predict 12 distinct diabetes types and prediabetic. The diabetes types are namely: Type 1, Type 2, Type 3c, Gestational, MODY, LADA, Secondary, Neonatal Mellitus, Wolcott-Rallison Syndrome, Steroid-Induced, Cystic Fibrosis-Related, and Wolfram Syndrome.

**Fig. 3:**
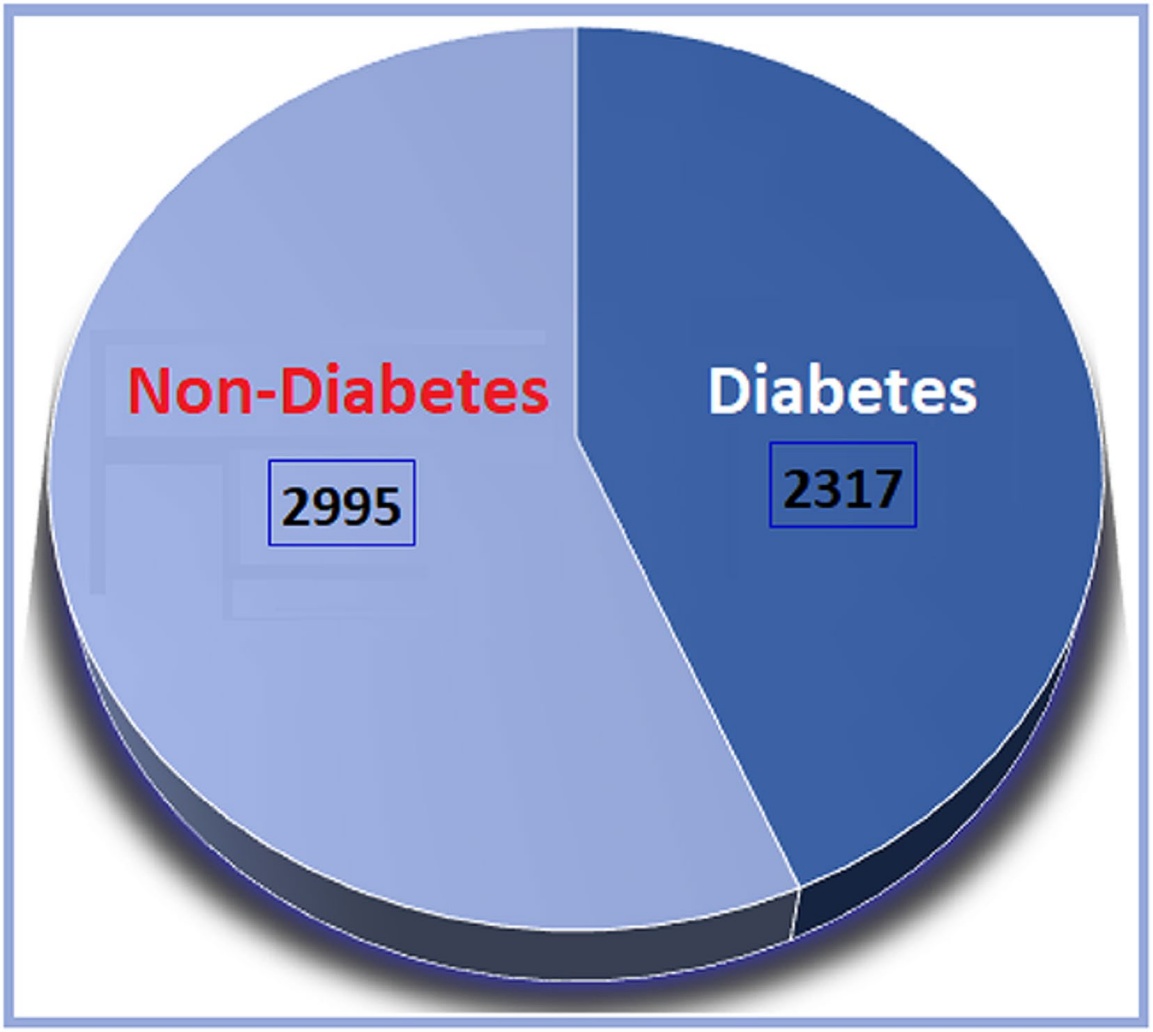
The number of diabetes and non-diabetes patients.

**Fig. 4:**
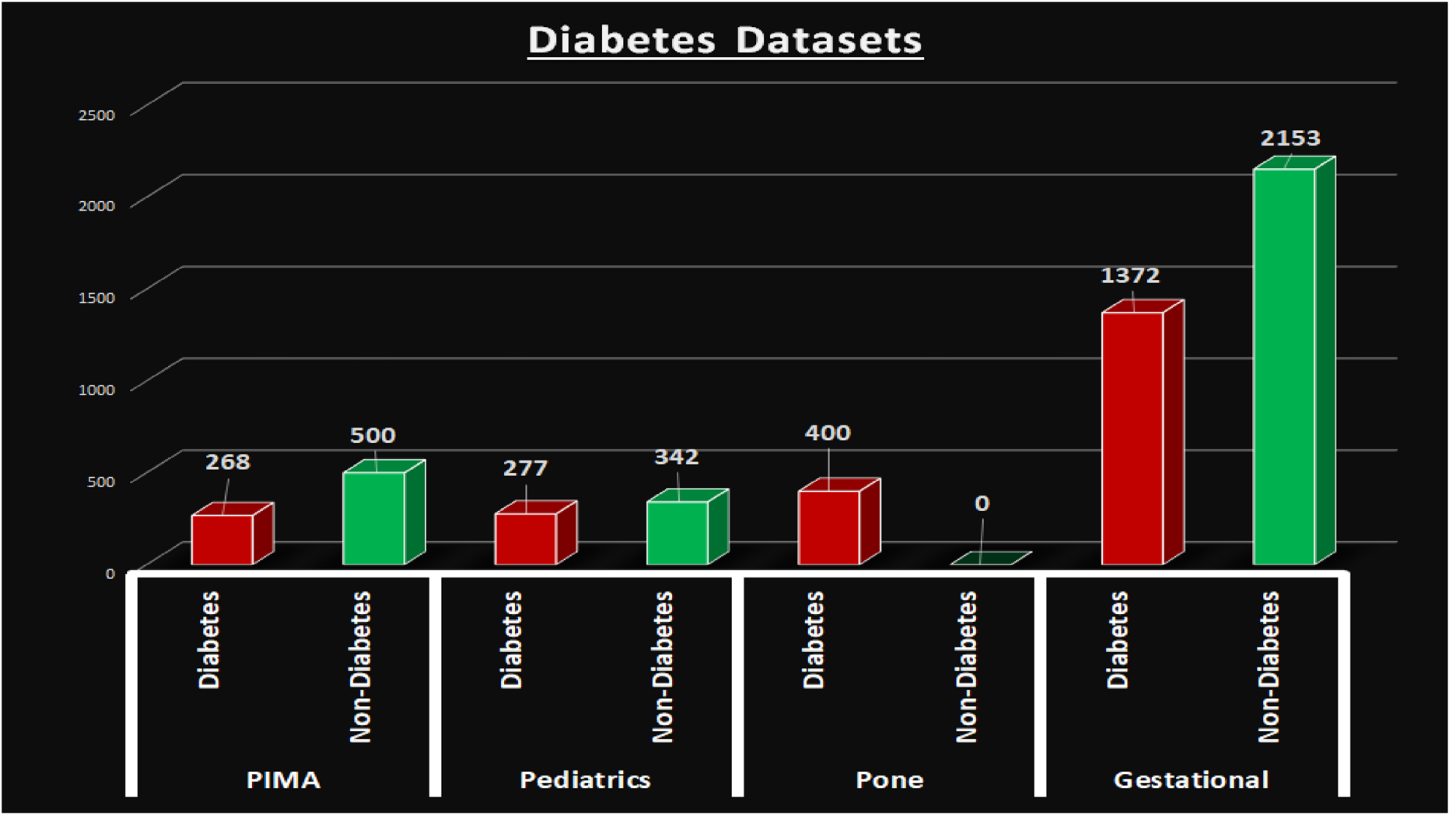
The PIMA, pediatrics, CS, and gestational diabetes datasets.

Figure 3 shows the binary distribution of diabetes (2317 diabetic and 2995 non-diabetic patients), while Fig. 4 outlines the composition of the four source datasets. Additionally, a rich Kaggle diabetes dataset covering various forms of the disease—such as Steroid-Induced, Neonatal, Prediabetes, Type 1, and Wolfram Syndrome—was referenced to support broader analysis of genetic, lifestyle, and medical factors contributing to diabetes.

**Fig. 5:**
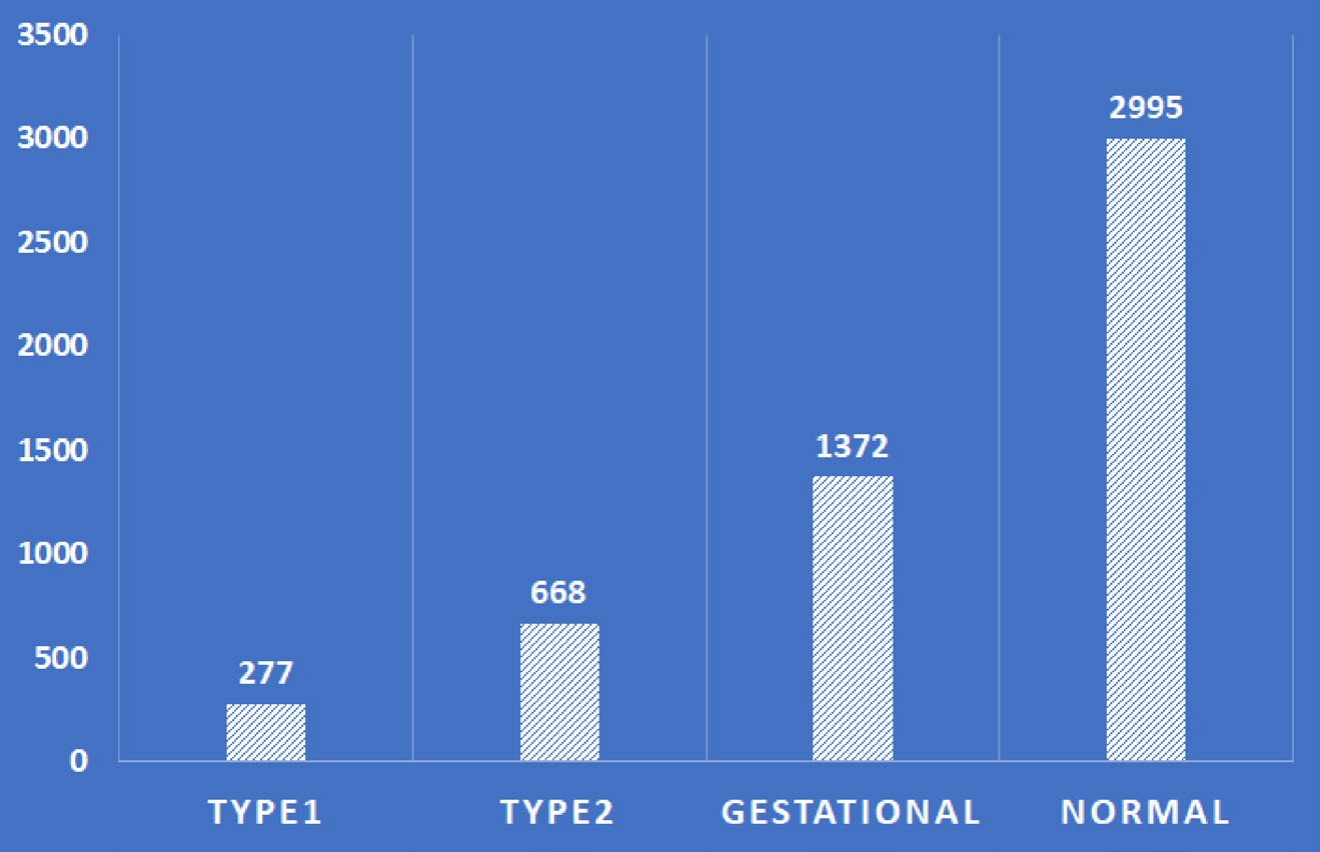
The diagnosis types.

Table 1 presents the DTD dataset, which comprises thirteen attributes and their comprehensive descriptions. The diagnosis attribute serves as the dependent output variable, while the remaining thirteen attributes are considered independent input features. The DiagnosisType label is utilized for multiclassification purposes, as depicted in Fig. 5.

**Table 1:** The attribute of the DTD dataset.

Attribute	Description
Age	Age (Years)
Sex	Gender (Male/Female)
BPressure	Diastolic blood pressure (mmHg)
NPregnancies	Number of times pregnancy
BMI	Body mass index (kg/m^2^)
HbA1c	Glycosylated haemoglobin (mmol/mol)
Insulin	Two-hour serum insulin Level (μU/mL)
FOGTT	Fast oral glucose tolerance test (mmol/L)
OGTT	Post-prandial oral glucose tolerance test (mmol/L)
PGlucose	Post-prandial plasma glucose level after 2 h (mmol/L)
FGlucose	Fasting plasma glucose level (mmol/L)
Diagnosis	Diabetes diagnosis 1 for a positive test and 0 for a negative test (diabetes/non-diabetes)
DiagnosisType	Diagnosis output Types (normal, type1, type2, or gestational)

### Data preprocessing

Some errors can result from human mistakes during the previous data collection phase. This led us to perform a preprocessing step that included adjusting the data format, handling missing values, feature selection, data sampling, and feature scaling.

#### Data format

The current phase is concerned with manipulating the collected input attributes to be in a clear and correct format. The preprocessing phase is used to organize and clean the data for further analysis and processing. This approach assists in the accuracy and precision of interpreting data features via the ML classifier algorithms. We prepared the DTD dataset in CSV file format.

#### Missing values

**Fig. 6:**
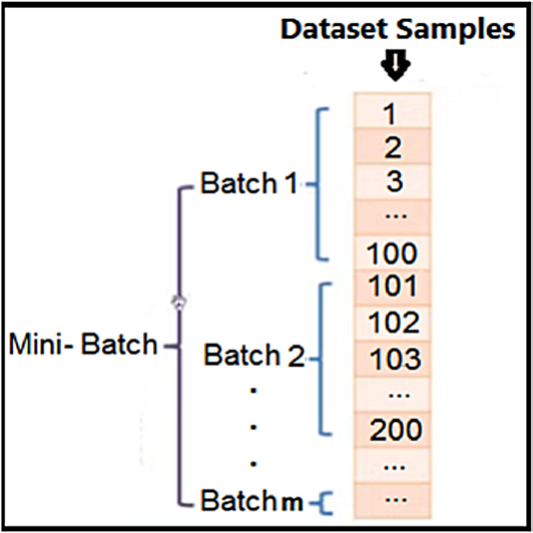
The dataset mini-batches.

An empty value within the attributes of the DTD dataset indicates a missing value, which is typically denoted by null indicators. Missing data samples may arise from errors during the data collection phase or from unperformed analysis requests. Such missing values can detrimentally impact the overall performance of the system^25^. In patient records, missing values may occur for one or multiple attributes for a defined percentage of patients. Addressing the issue of missing values can be approached in two ways. First, one may opt to eliminate features with missing values, although this risks discarding pertinent information and reducing the dataset size. Second, one can replace missing values by Multiple Imputation by Chained Equations (MICE) for the DTD dataset^26^. MICE is widely used to handle missing data in datasets that contain both numerical (continuous) and categorical (discrete) variables. The imputation used in MICE is a regression-based model where each missing value Yj is predicted using the observed values of other variables^27^. The general form for imputation is indicated using Eq. (1):

1 $$Y_{j}^{m} = f\left( {X^{m} ,\theta_{j} } \right)$$

where ${\mathrm{Yj}}^{m}$ is the imputed value of variable j in the m-th imputed dataset, Xm represents the observed values of all other variables in the dataset for the m-th imputed dataset. f is a regression model (e.g., linear regression or LR) that predicts ${\mathrm{Yj}}^{m}$ based on X^m^. The structure of the DTD dataset multiclass classification includes numerical features like Age, BMI, BPressure, NPregnancies, HbA1c, FGlucose, PGlucose, FOGTT, POGTT, Insulin, and categorical features (e.g., Sex, Diagnosis). The target classes (e.g., Normal, Type 1, Type 2, or gestational diabetes). A sample of the DTD dataset is depicted in Fig. 6. where missing values are indicated by white cells. The number of missing values in the DTD dataset is detailed in Table 2. Then we standardize the data using Standard Scaler.

**Table 2:** The missing values of The DTD dataset.

Attribute	Null Count
Age	406
Sex	402
BPressure	2350
NPregnancies	558
BMI	1354
HbA1c	4488
Insulin	4824
FOGTT	4802
POGTT	1027
PGlucose	1257
FGlucose	4673
Diagnosis	0
DiagnosisType	0

#### Handle class imbalance

We use the Synthetic Minority Over-sampling Technique) SMOTE) A technique to balance imbalanced datasets by creating synthetic samples of the minority class^35^. Instead of duplicating rows, it generates new data points using interpolation. It’s used before training classifiers to prevent the model from being biased toward the majority classes. SMOTE helps by equalizing the number of samples per class and improving model generalization across all classes. The DTD samples are distributed as follows:—Normal patients are: 3003, Type 1 patients are: 277, Type 2 patients are: 659, and Gestational patients are: 1373. Using the SMOTE technique, we balance the dataset to be: Normal patients are 3004, Type 1 patients are 3004, Type 2 patients are 3004, and Gestational patients are 3004, to prevent overfitting.

#### Feature selection

Feature selection plays a crucial role in the feature extraction process, aiming to eliminate redundant features and retain those essential for constructing an efficient predictive model, thereby enhancing classification accuracy. This process aids in comprehending the significance of all extracted features, allowing for the utilization of valuable values while discarding outlier features. By prioritizing features with the highest correlation and importance scores, the feature selection method minimizes the execution time and mitigates the risk of data overfitting. Identifying interactions among input variables that influence system output performance is of paramount importance.

To analyze correlations between different attribute types (numerical and categorical), we apply two statistical techniques One-Way ANOVA test and the Chi-square tests. The ANOVA test is used to compare the means of a numerical variable across multiple categories of DiagnosisType (target classes). ANOVA checks if the mean Age, BMI, BPressure, NPregnancies, HbA1c, FGlucose, PGlucose, FOGTT, POGTT or Insulin differs significantly across DiagnosisType. It helps to assess whether the variation in the data is due to differences between groups or if it’s just random variation. To perform ANOVA test, state the null hypothesis (H₀): The means of the groups are equal, using Eq. (2):

2 $${\mathrm{H}}_{0} : \, \mu_{{1}} = \, \mu_{{2}} = \, \mu_{{3}} = \, \mu_{{4}} = \ldots \ldots . \, \mu_{{\mathrm{k}}}$$

where k is the total number of groups.

Alternative hypothesis (H₁) at least one of the group means is different from the others using Eq. (3):

3 $${\mathrm{H}}_{1} {:}\mu_{{\mathrm{i}}}$$

where I ∈ {1, 2, 3, …, k}.

The F-statistic (F) is calculated by comparing the variance between groups and within groups using Eq. (4):

4 $${\mathbf{F}} = \frac{{{\mathbf{Variance}} {\mathbf{between}} {\mathbf{Groups}} }}{{{\mathbf{Variance}} {\mathbf{within}} {\mathbf{Groups}}}}$$

We use the p-value statistical measure that helps to determine the significance of your results in a hypothesis test. It tells the probability of obtaining results at least as extreme as the observed results, if the null hypothesis (H_0_) is true. All the p-values are very small (< 0.0001), with many being close to zero, indicating that there is a statistically significant difference in Age, BPressure, NPregnancies, BMI, HbA1c, POGTT, FOGTT, PGlucose, FGlucose, and Insulin across DiagnosisType as indicated in Table 3.

**Table 3:** F-statistic and *p* value of numerical attributes.

ssFeatures	F-statistic	*p* value
Age	857.0862052972869	< 0.0001
BPressure	741.3978375145017	< 0.0001
NPregnancies	128.9466926504602	1.2597027730130282e-80
BMI	772.8786519364006	< 0.0001
HbA1c	1449.667114228026	< 0.0001
POGTT	1427.3873303973114	< 0.0001
FOGTT	1077.4578912003146	< 0.0001
PGlucose	812.2141420165092	< 0.0001
FGlucose	645.818181641019	< 0.0001
Insulin	239.01145651388003	1.8068349089626406e-145

We also apply the Chi-Square Test (χ^2^) to evaluate whether two categorical variables are statistically associated. This test is especially suitable when you want to examine if DiagnosisType is related to categorical features like Sex or Diagnosis. In practice, the test compares each observed cell frequency with its expected count under the null hypothesis of independence. The Formula for the Chi-Square Test using Eq. (5):

5 $$X^{2} = \sum \frac{{\left( {O - E} \right)^{2} }}{E}$$

where O is the actual count in each category, E is the expected count if there is no association.

The degrees of freedom (D) using Eq. (6):

6 $${\text{D }} = \, \left( {{\mathrm{R}} - {1}} \right){\text{ x }}\left( {{\mathrm{C}} - {1}} \right)$$

where R is the number of rows and C is the number of columns.

The Chi-square statistic is notably large, and the p-value is less than 0.0001 well below the conventional threshold of 0.05, indicating a significant association between the Sex and DiagnosisType variables. Similarly, the Chi-square between Diagnosis and DiagnosisType is also very large, with a p-value less than 0.0001, confirming a significant association, as shown in Table 4.

**Table 4:** Chi-square, *P* value, and degrees of freedom of sex and diagnosis with diagnosistype.

	Chi-square statistic	*P* value	Degrees of freedom
Sex	1752.5138392719468	< 0.0001	3
Diagnosis	5307.930910834319	< 0.0001	1

#### Data sampling

We partition the training dataset into smaller chunks. This chunk of the DTD dataset is divided into smaller batches aids in the training phase, as illustrated in Fig. 6.

#### Train phase

**Fig. 7:**
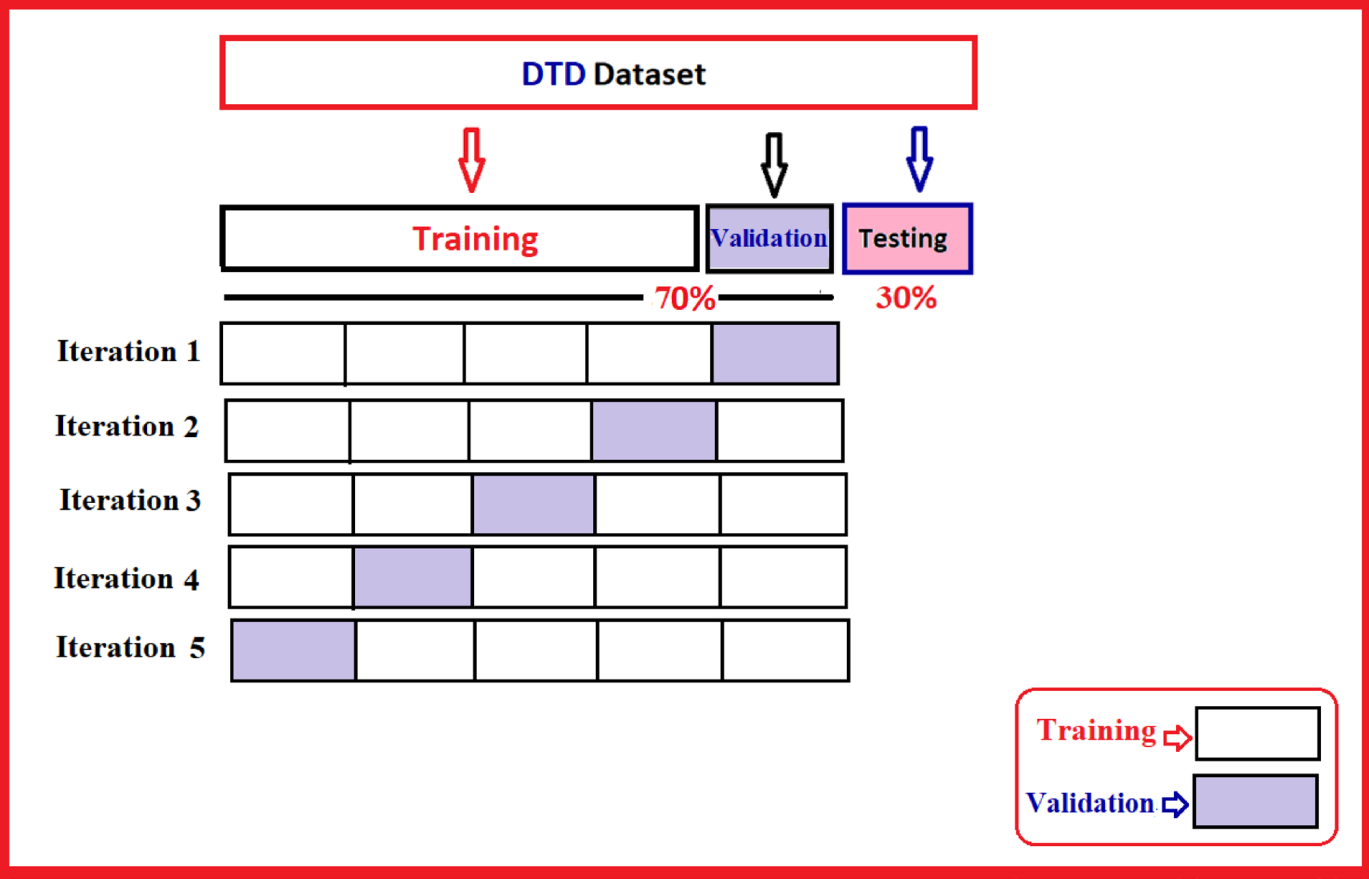
The train, validation and test five k-folds.

This phase focuses on training multiple classifiers using a set of input attributes prepared during the data preprocessing stage. Classifiers such as ANN, LR, NB, DT, AB, RF, GB, KNN, and ANN are each individually trained on the dataset. The objective is to build models capable of accurately classifying unseen data into one of four categories: Type 1 Diabetes, Type 2 Diabetes, Gestational Diabetes, or Normal based on the predefined diagnosisType label. The dataset, containing 5312 records, is split into two primary subsets: 70% (3718 samples) for training and 30% (1,594 samples) for testing, as illustrated in Fig. 7. The training set is further subjected to 5 fold cross-validation to enhance model reliability^34^. In each fold, approximately 56% of the total dataset is used for training, while about 14% is used for validation. This process is repeated five times, with each fold serving as the validation set once. Stratified sampling is applied during splitting to maintain balanced class distributions. The test set remains untouched throughout the cross-validation process and is used only for final performance evaluation.

#### Machine learning multi-class classification

The pivotal aspect of the multiclass classification process is training the classifier. This step involves creating a model by training it with nine classifiers, which will subsequently be utilized to classify unlabeled “DiagnosisType” into the four output categories Normal, Type 1, Type 2, Gestational patients. We proceed by training nine ML classifiers, including ANN, LR, NB, DT, AB, RF, GB, ET and KNN^34^. These supervised ML algorithms are specifically chosen to perform multiclassification on the DTD dataset. Following this, the nine ML classifiers are compared, and the most suitable ones for the dataset are selected. The output of this phase is a trained classifier referred to as a model, which is prepared for testing. The parameters are fine-tuned on the developed predictive model, and performance measures are calculated, resulting in the construction of a superior ML model with a highly accurate performance level.

#### ANN classification algorithm

**Fig. 8:**
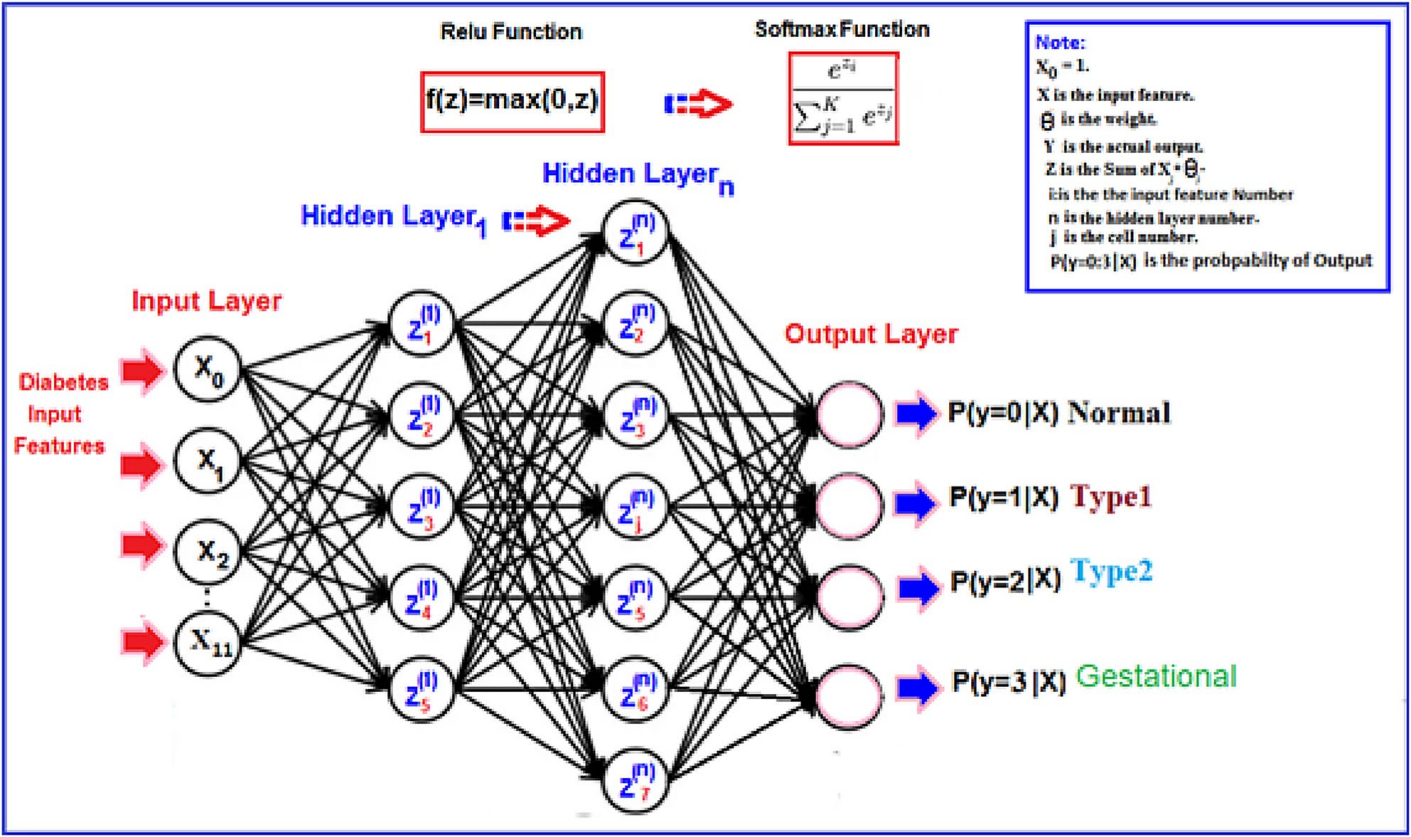
ANN architecture.

Previously, we conducted binary classification to distinguish between diabetic patients and non-diabetic patients. Subsequently, in our proposed model, we utilized nine supervised ML techniques to perform multiclassification of diabetes type prediction using an ANN. An ANN typically comprises multiple layers of units known as neurons. In each mini batch, the input features from the DTD dataset are forwarded to the initial input layer. The neurons in the first layer receive a vector composed of twelve input features, while the subsequent hidden layer’s neurons are linked to the input layer’s neurons through a combination of weights and the Rectified Linear Unit (ReLU) activation function^37^. The neurons in the last output layer receive a combination of outputs with corresponding weights and apply the SoftMax activation function. The ANN is configured for four output nodes: normal patient, Type 1, Type 2 and gestational diabetes patients as shown in Fig. 8. The cross-entropy of the multinomial distribution serves as the cost function, measuring the disparity between predicted and actual outputs to adjust weights and biases accordingly. We use the swarm optimization for fine-tuning hyperparameters^36^. The ADAM algorithm is employed to update the assigned weight and bias values, with a regularization factor of 0.01 and 50 epochs, respectively. The number of iterations needed to complete one epoch corresponds to the number of batches. This iterative process continues until the desired output aligns closely with the actual output. The SoftMax function aids in the final classification with multiple output probabilities (0, 1, 2, or 3) for different patients, as depicted in Fig. 10. With this, our training concludes, yielding a multiclassification prediction with high accuracy. The ANN Algorithm is described in online Appendix B.

**Fig. 9:**
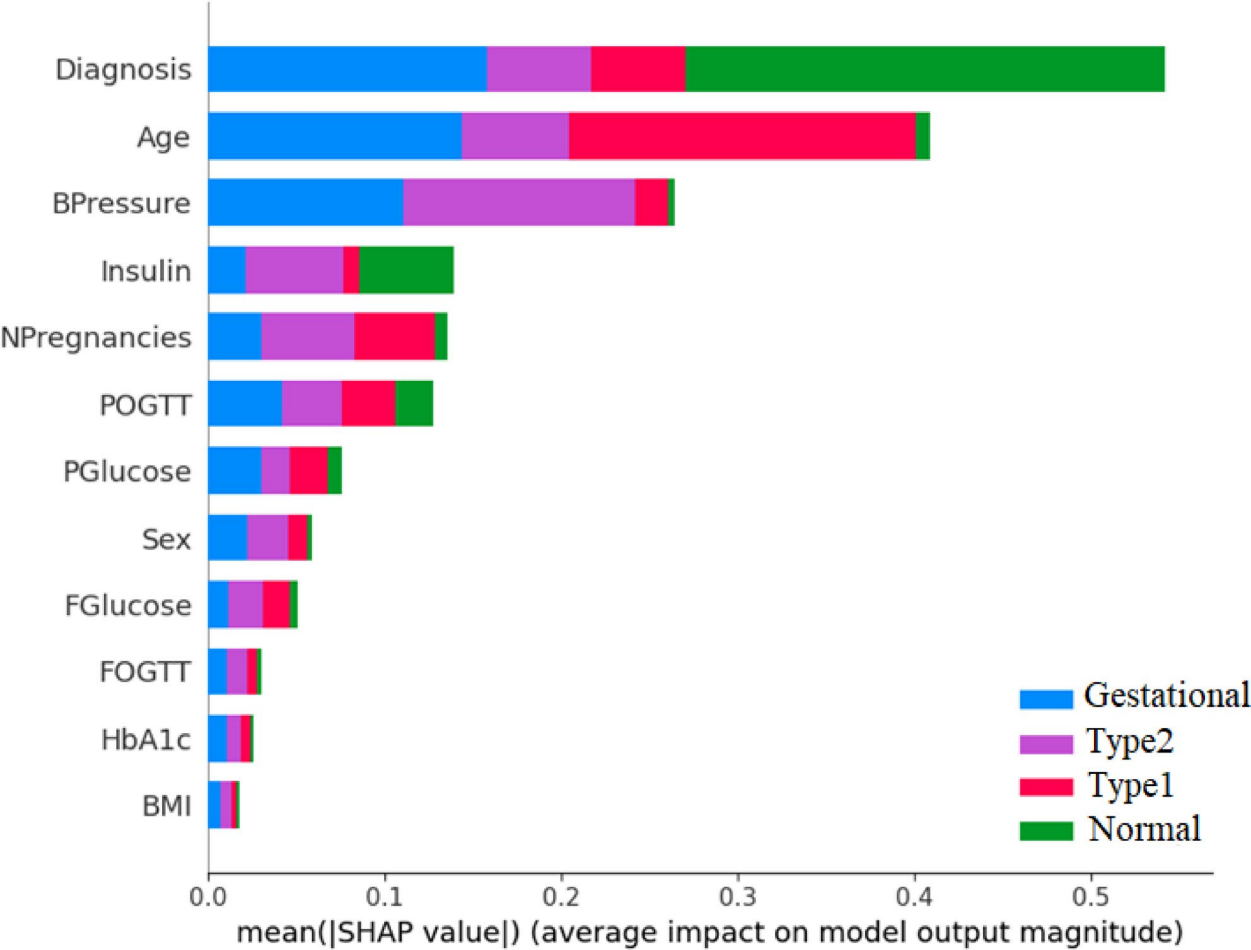
The SHAP of DTD dataset.

**Fig. 10:**
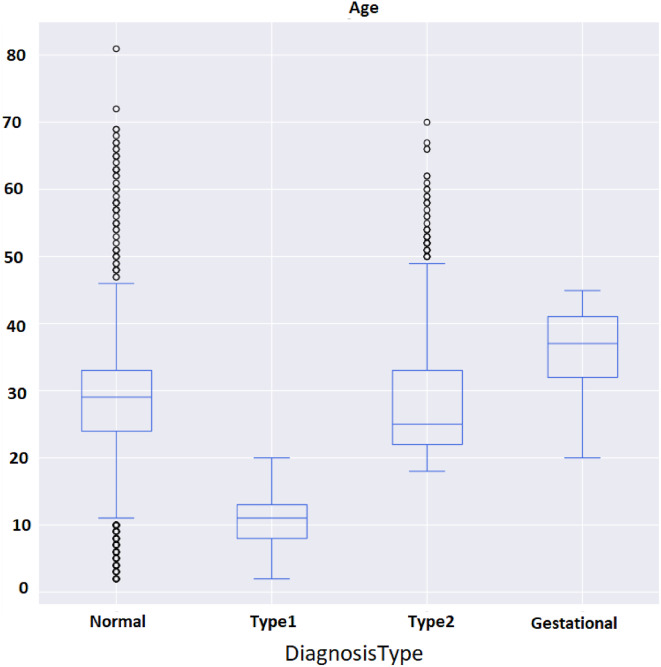
Age and diagnosis type attributes.

#### Test phase

The multiclassification technique was subsequently applied to accurately assign a class label to unlabelled DiagnosisType, effectively distinguishing between Type 1, Type 2, Gestational diabetes, and normal cases. A mapping function is utilized to classify the unlabelled DiagnosisType and ascertain its appropriate label. It is imperative to compute the probability of assigning the patient to the respective class label using eight multiclassification techniques.

#### Optimization

We utilize the Keras and TensorFlow libraries to construct a model for the ANN. The training–testing split and cross-validation functions from the Sci-Kit-Learn library are employed for data splitting. Various ML algorithms, including LR, NB, DT, AB, RF, GBC, ET, KNN, and ANN, are implemented in this phase. The primary objective is to enhance multiclassification efficiency, accuracy, and reduce computational complexity compared to manual methods. The ANN model is trained with tuned hyperparameters using the Particle Swarm Optimization (PSO) technique to determine the best-fit probable value for model optimization. PSO plays a crucial role in improving the model’s performance by adjusting the particles’ positions in the swarm to find the optimal set of parameters. PSO optimizes several key parameters, such as the number of hidden units, batch size, learning rate, momentum, and the number of hidden layers. In this case, PSO will search for the optimal number of neurons. The batch size, which determines the number of training examples processed in each iteration before updating the model’s weights, will be optimized between 5 and 64. PSO also searches for the best learning rate (lr) within the range of 0.0001 to 0.1, using Adam optimizer to minimize output error during backpropagation, which controls how quickly the model updates its weights during training.

#### Evaluation

This phase focuses on evaluating the performance of each classifier by comparing predicted class labels with the actual labels using the trained models. To interpret and understand model behaviour, we employ SHapley Additive exPlanations (SHAP) Summary Plots, which illustrate the contribution of each feature to individual predictions as well as the overall model output as shown in Fig. 9. These plots enhance transparency by highlighting which features influence predictions positively or negatively, thereby improving interpretability and trust in the model. Additionally, we apply Particle Swarm Optimization (PSO) for hyperparameter tuning to optimize each classifier’s performance. The effectiveness of the machine learning algorithms is assessed using various evaluation metrics, including precision, Mean Squared Error (MSE), R^2^ score, training accuracy and AUC^3^.

#### Prediction

The performance of all classifiers is compared to determine the most effective model for recommendation. The selected classifier demonstrating the highest predictive accuracy serves as the optimal model, significantly enhancing the system’s overall capability to predict class labels for new data. To support this evaluation, three key visualizations are generated: a Confusion Matrix applied to the full dataset, ROC Curves for each class to assess classification performance, and a Learning Curve that illustrates the relationship between training size and both training and validation accuracy. Finally, the best model is used to predict the DiagnosisType on an external dataset namely “diabetes_prediction_dataset”. The proposed system is designed to use a trained and optimized classifier to make predictions on a new, external dataset that was not part of the original training data. It first loads the external dataset from a CSV file, removes the target column if it exists (since the goal is to predict it), and ensures that the data’s structure matches the training features. It then applies the same preprocessing steps used during training such as imputing missing values and standardizing the data to prepare it for prediction. Using the final model, which was optimized through PSO, it generates predictions for each sample, mapping the predicted numeric labels to meaningful class names like “Normal,” “Type1,” “Type2,” or “Gestational.” Finally, the results, including the original features and predicted labels, are saved to a new CSV file for review or further analysis following the procedure outlined in Algorithm 1. 

**Algorithm 1:**
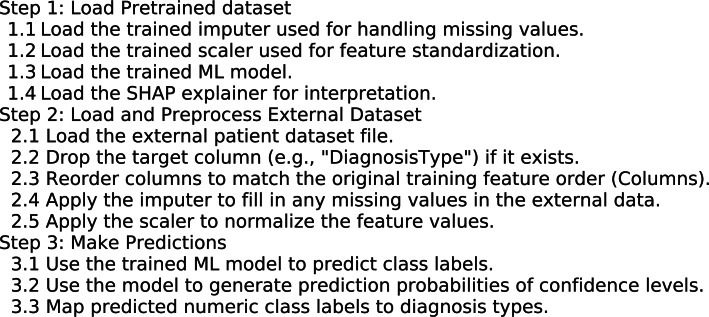
Predict and Interpret Using Trained ML Model

## Results and discussion

**Fig. 11:**
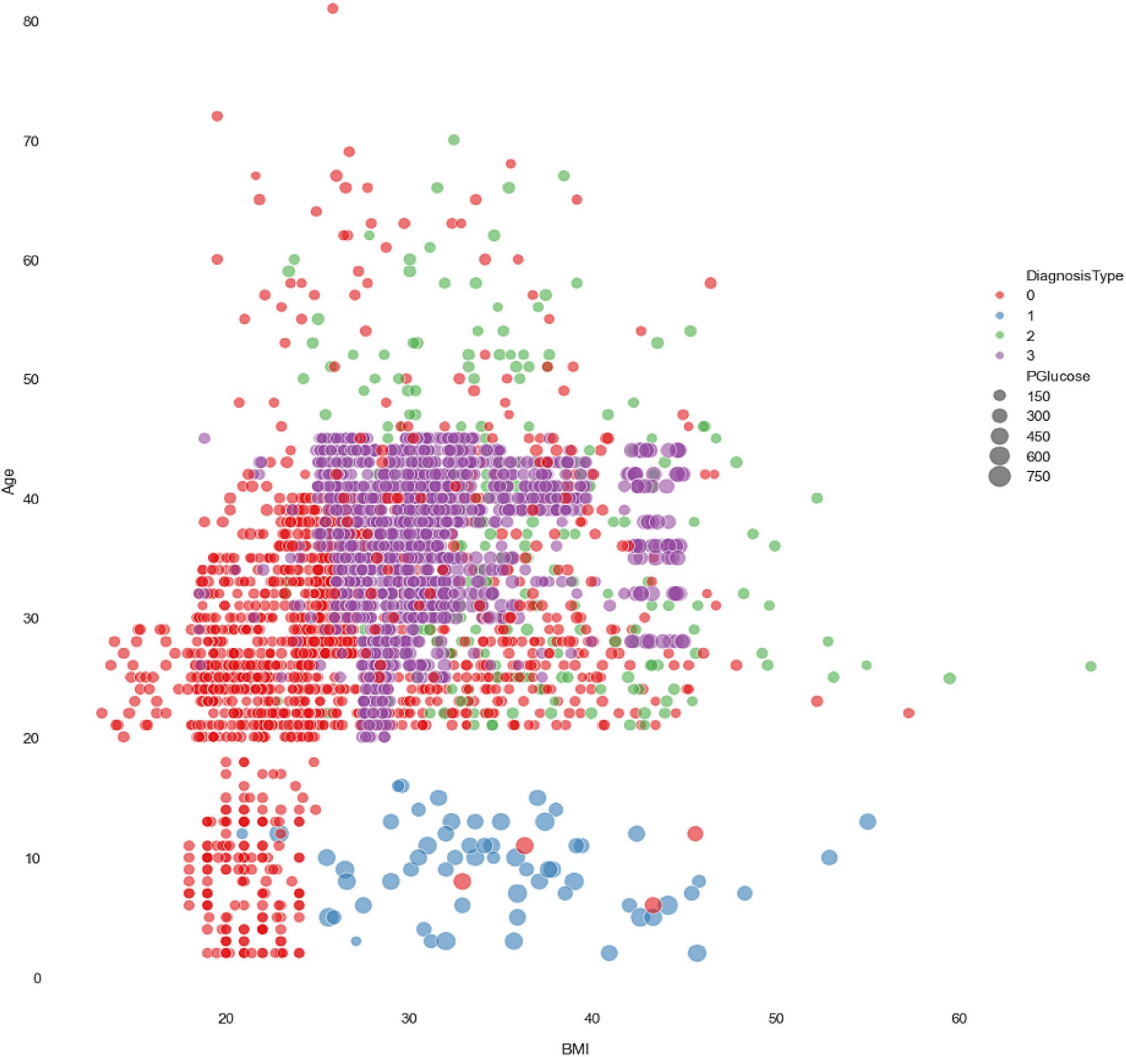
Age and BMI group with diagnosis type and PGlucose.

**Fig. 12:**
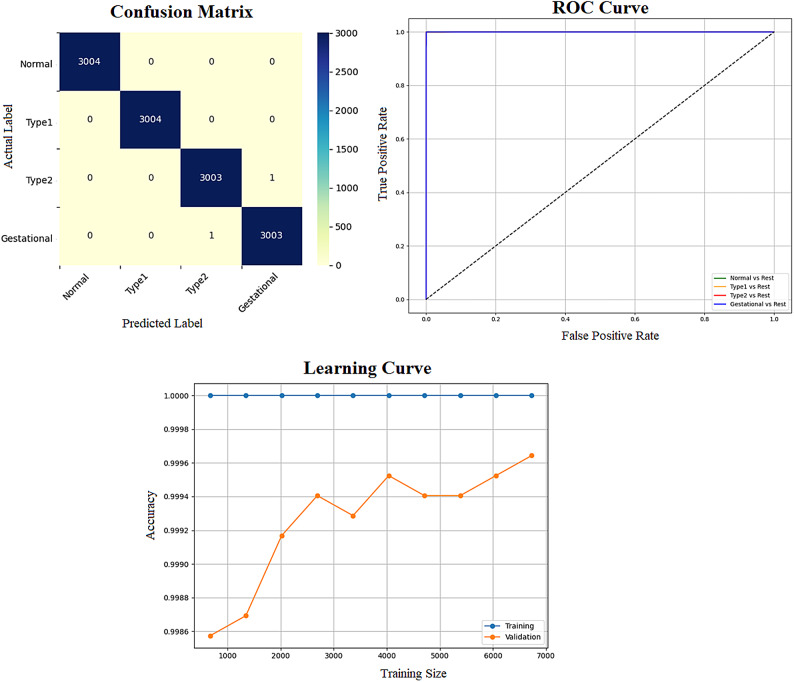
RF Confusion Matrix, Multiclass ROC Curve, and Learning Curve.

**Fig. 13:**
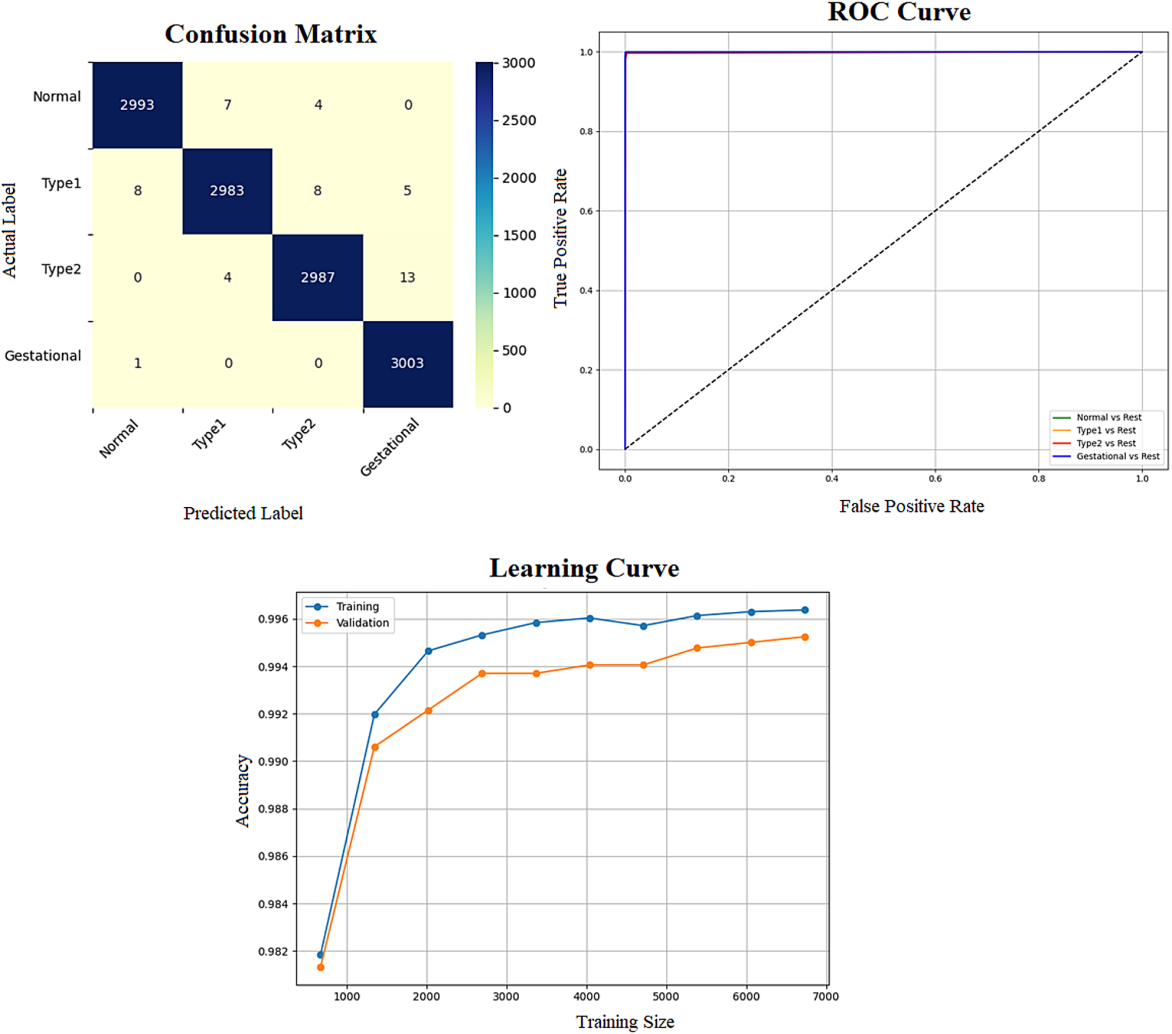
KNN Confusion Matrix, Multiclass ROC Curve, and Learning Curve.

**Fig. 14:**
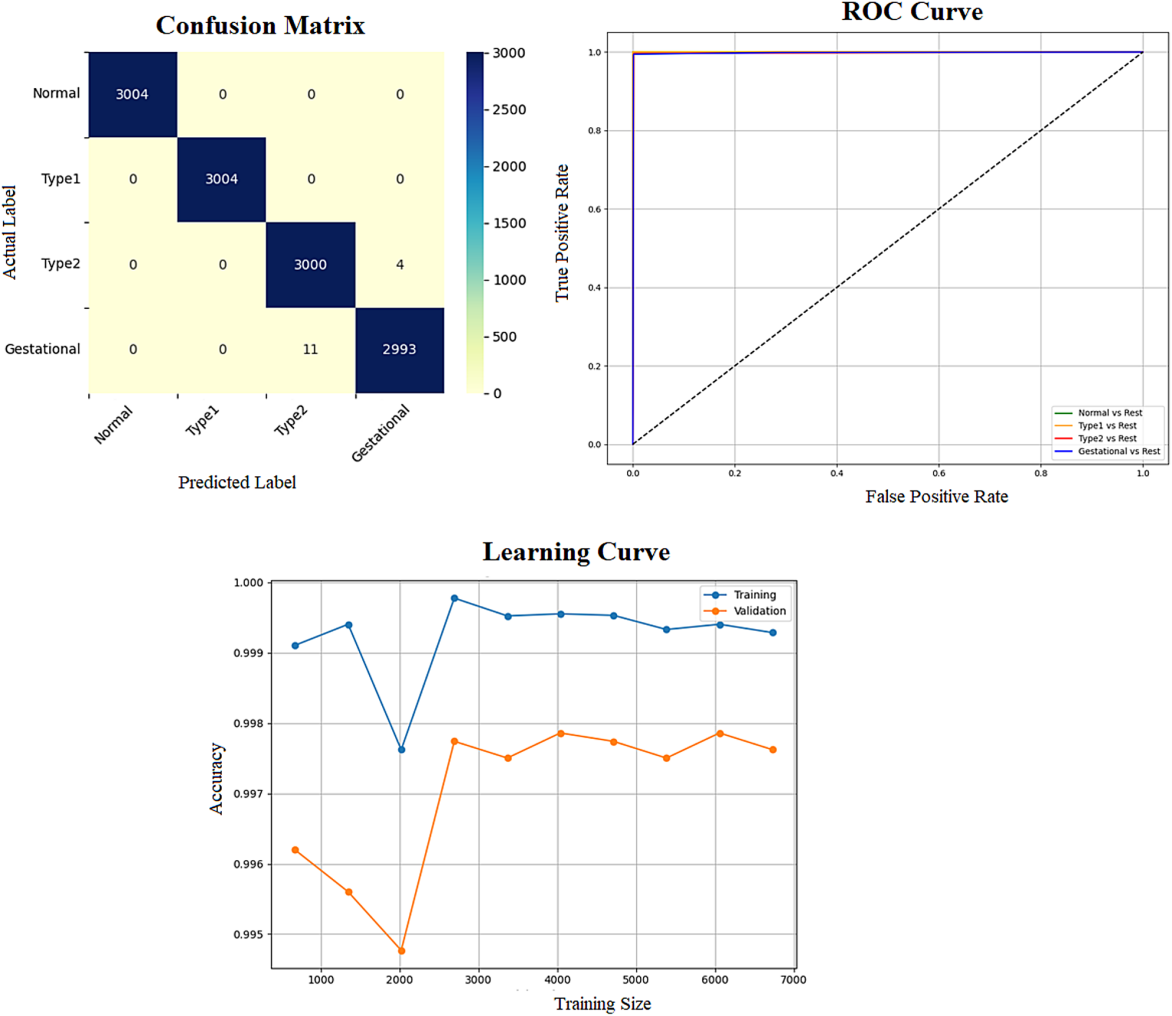
DT Confusion Matrix, Multiclass ROC Curve, and Learning Curve.

**Fig. 15:**
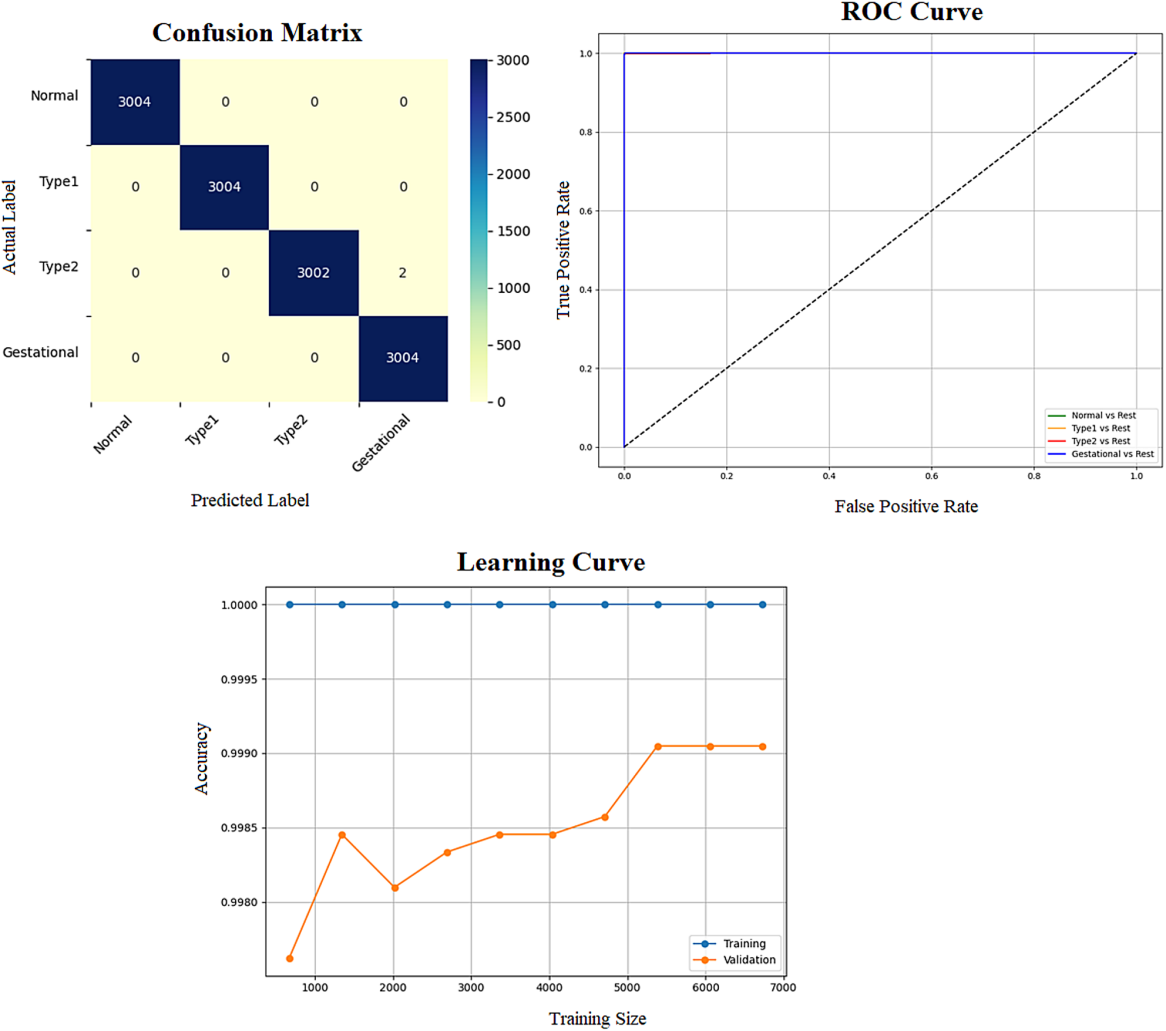
GB Confusion Matrix, Multiclass ROC Curve, and Learning Curve.

**Fig. 16:**
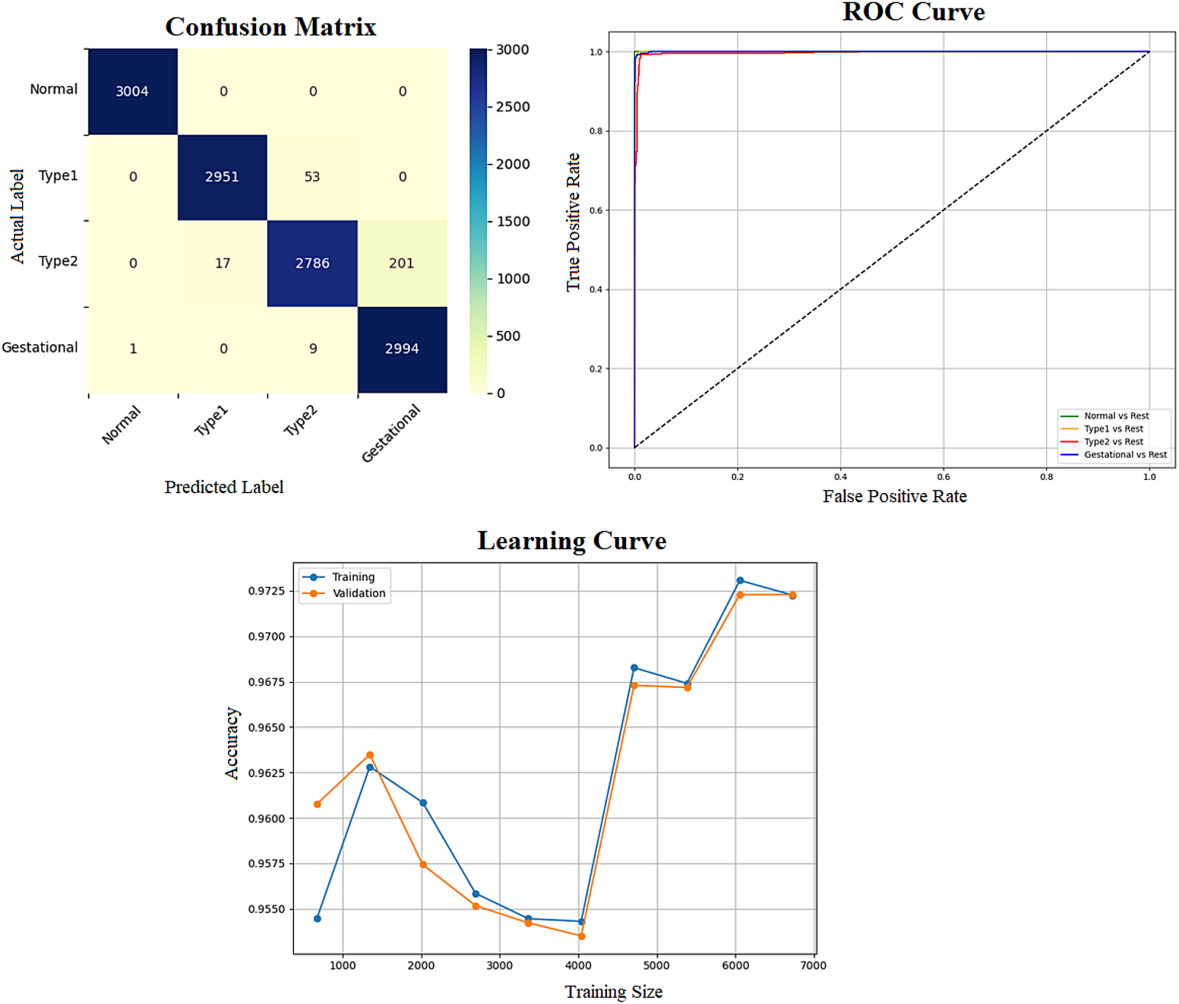
NB Confusion Matrix, Multiclass ROC Curve, and Learning Curve.

**Fig. 17:**
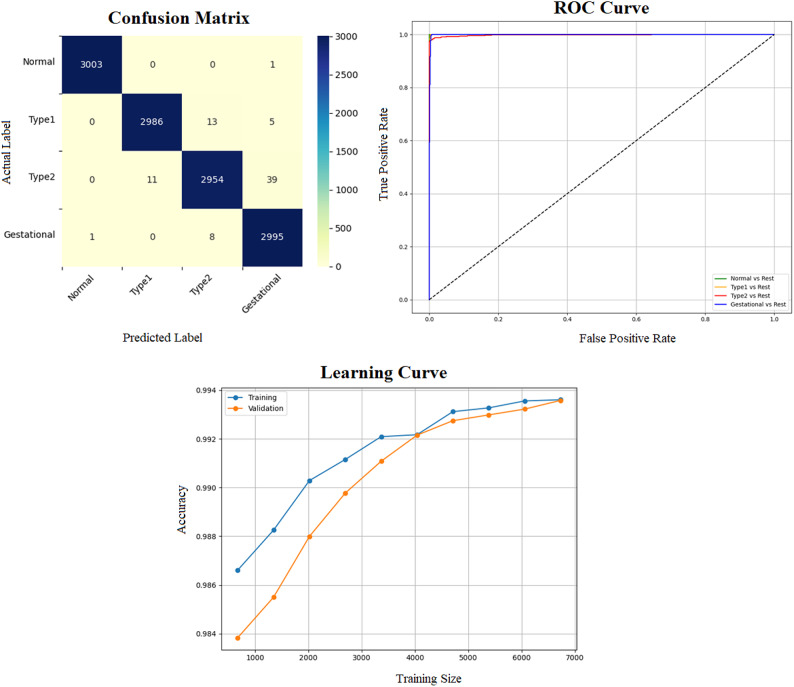
AB Confusion Matrix, Multiclass ROC Curve, and Learning Curve.

**Fig. 18:**
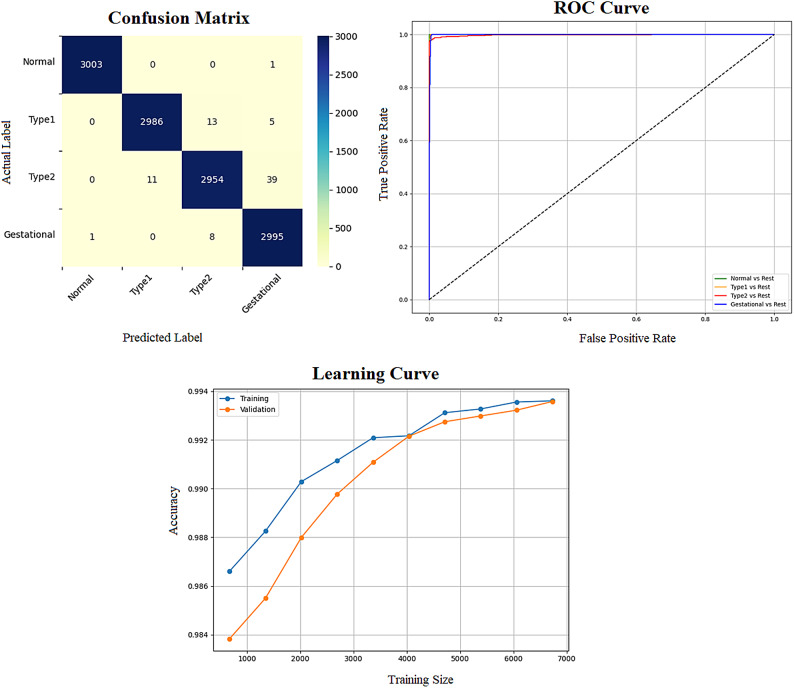
LR Confusion Matrix, Multiclass ROC Curve, and Learning Curve.

**Fig. 19:**
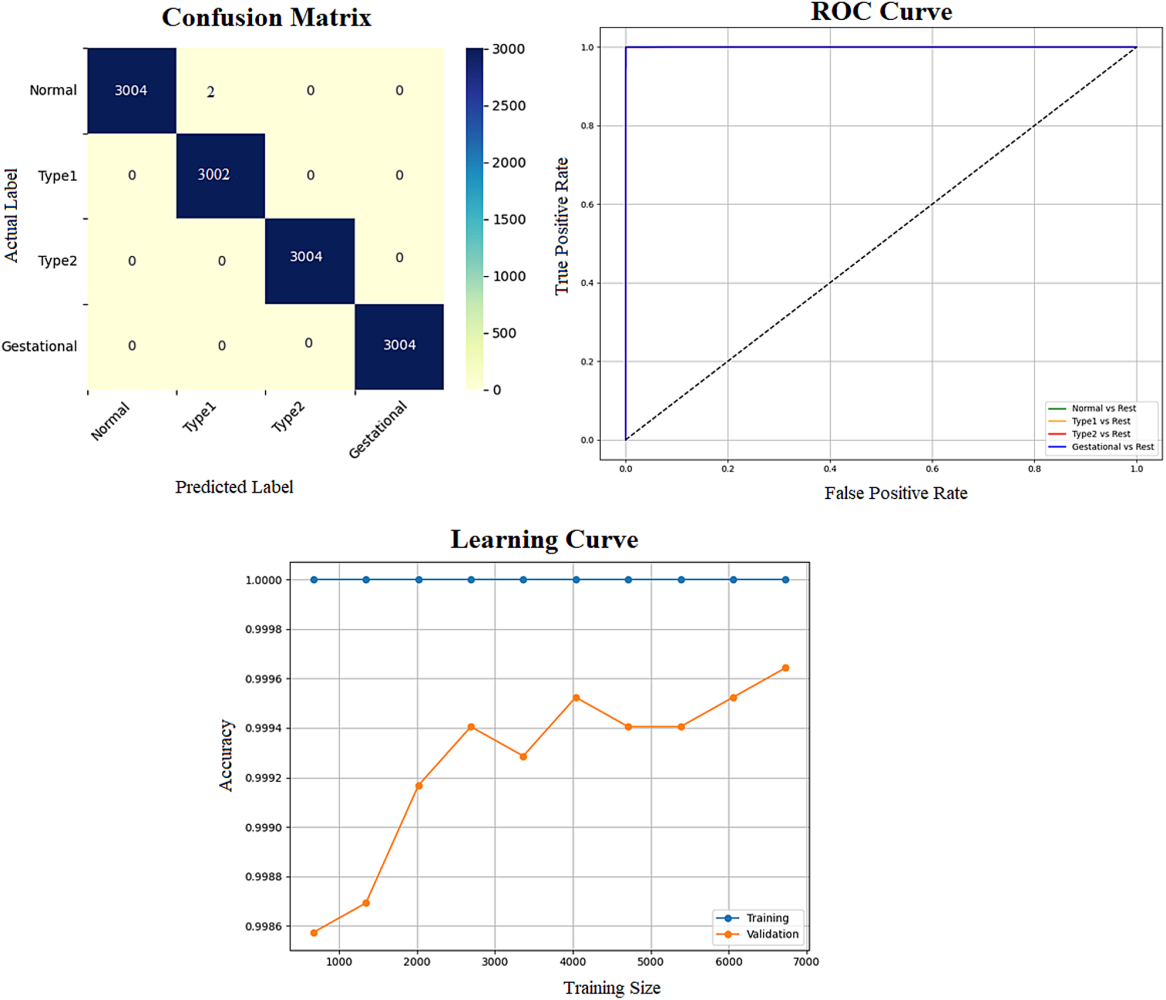
ANN Confusion Matrix, Multiclass ROC Curve, and Learning Curve.

**Fig. 20:**
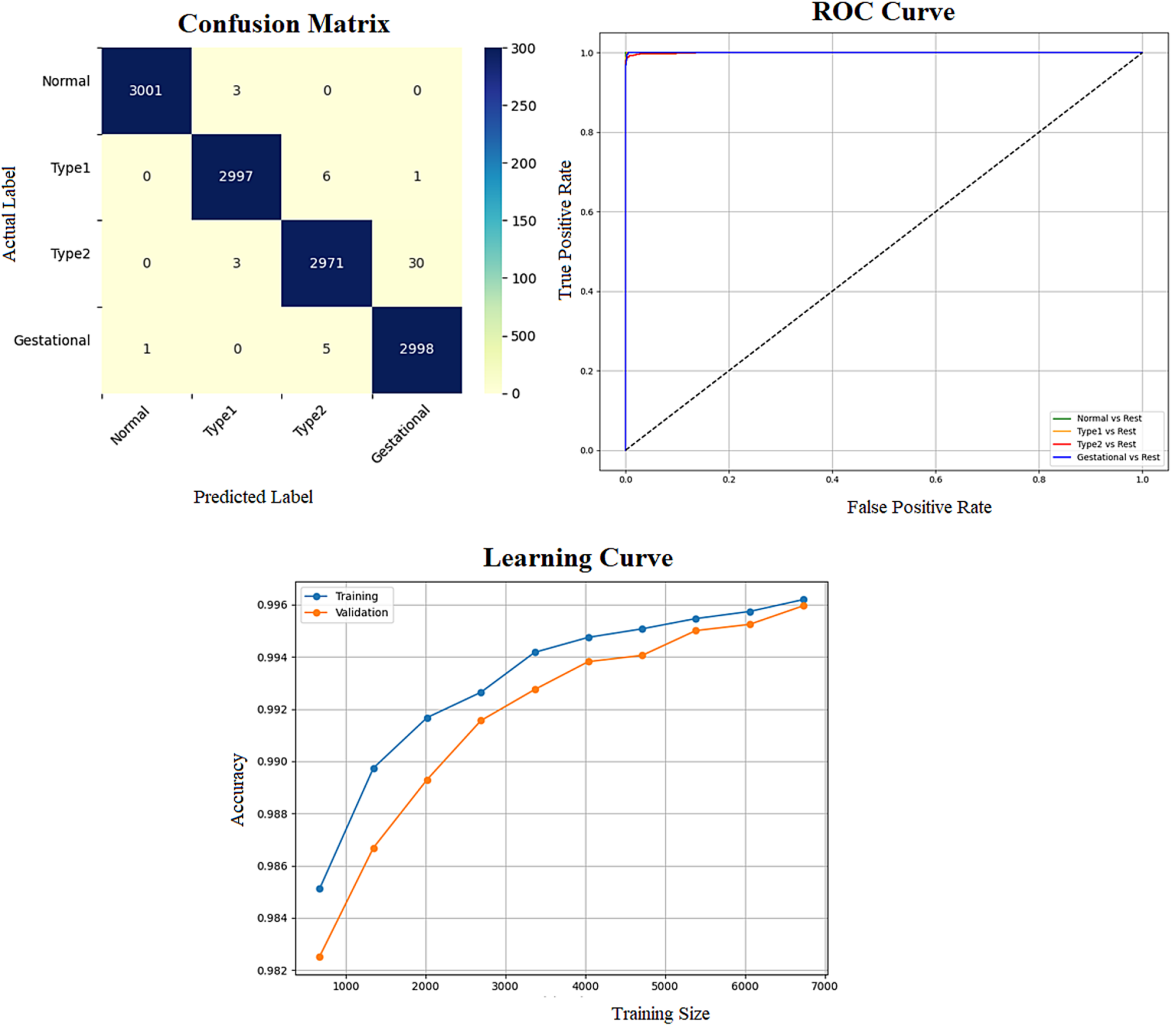
SVM Confusion Matrix, Multiclass ROC Curve, and Learning Curve.

Early detection of diabetes significantly improves clinical outcomes and facilitates effective long-term disease management. In this study, a supervised ML system was developed to classify multiple types of diabetes. The proposed ML model is designed for seamless integration into routine clinical workflows and self-management protocols, supporting both diagnosis and risk assessment of various diabetes types. The model was trained and evaluated on the DTD dataset, a composite dataset that integrates patient records from four distinct sources: Pediatrics, PID, Pone, and Gestational Diabetes datasets. This heterogeneous dataset enabled a multiclass classification task covering four categories: Type 1 Diabetes, Type 2 Diabetes, Gestational Diabetes, and Normal (non-diabetic). A comprehensive preprocessing phase was conducted to prepare the dataset for modelling. This included standardization of file formats, removal of outliers and inconsistencies, and imputation of missing values using the MICE (Multivariate Imputation by Chained Equations) technique, applied to both numerical and categorical features. To address class imbalance within the dataset, the SMOTE (Synthetic Minority Over-sampling Technique) method was used, generating synthetic samples for underrepresented classes and improving classifier fairness. Nine supervised ML algorithms were employed for classification: LR, NB, DT, RF, AB, GB, SVM, KNN, and ANN. To support feature analysis and model interpretability, several exploratory visualizations were generated. Figure 10 displays a grouped box plot showing the relationship between Age and DiagnosisType, while Fig. 11 presents a scatter plot of Age versus BMI, color-coded by DiagnosisType and scaled according to PGlucose values. These visualizations helped to confirm class separability and feature relevance, supporting the multiclass classification objective. To further improve classifier performance, each ML model underwent hyperparameter tuning using the PSO technique. PSO played a critical role by iteratively adjusting the positions of particles within the swarm to identify the optimal parameter configuration for each model. This process led to significant improvements in classification accuracy. To enhance model transparency, SHAP summary plots were generated. These plots quantified the contribution of each feature to both individual predictions and the overall model output, identifying the most influential variables and supporting clinical interpretability. In the final stage, the performance of all classifiers was compared to determine the most effective model for recommendation. The classifier achieving the highest predictive accuracy was selected as the optimal model, ensuring maximum generalization to new data. To support this selection, three critical evaluation visualizations were generated: a Confusion Matrix applied to the full dataset to assess class-wise prediction accuracy; ROC Curves for each class, using the One-vs-Rest (OVR) strategy to evaluate discriminatory power; and a Learning Curve illustrating training versus validation accuracy as dataset size increased. These are presented in Figs. 12, 13, 14, 15, 16, 17, 18, 19 and 20 and further detailed in online Appendix C. The selected model using the DTD dataset was used to make predictions on an external diabetes dataset namely diabetes_prediction_dataset include 9 features of 100,000 records. In addition, an external dataset, the diabetes_Dataset, comprising 34 features and 12 distinct diabetes types, is used in place of the previously employed DTD dataset to evaluate the system’s adaptability and generalization across a wider spectrum of diabetes classifications in online Appendix D. All stages of the pipeline were applied without modification, demonstrating the system’s flexibility and robustness when applied to more complex, real-world datasets. Model performance was assessed using multiple evaluation metrics, including Precision, Accuracy, MSE, R^2^ Score, and AUC. Among all classifiers, ANN demonstrated superior performance, achieving an overall classification accuracy of 99.98%. These models recorded minimal or no misclassifications across all classes and achieved the highest scores across all evaluation metrics.

The Eqs. (7–11) are used to measure the performance evaluation of the classification techniques as Precision Score, Recall Score and F1-Score parameters are calculated^28^. TN = True Negative, FP = False Positive, FN = False Negative, TP = True Positive. The precision score provides the accuracy of positive diabetes predictions using Eq. (7).

7 $${\text{Precision }} = \frac{{{\mathrm{TP}}}}{{{\text{TP }} + {\text{ FP}}}}$$

Recall score is the ratio of correctly predicted positive cases to all using Eq. (8).

8 $${\text{Recall }} = \frac{{{\mathrm{TP}}}}{{{\text{TP }} + {\text{ FN}}}}$$

F1-score is the weighted average of Precision and Recall using Eq. (9).

9 $${\text{F1 }} = \frac{{2{ * }\left( {\text{Precision * Recall}} \right)}}{{{\text{Precision }} + {\text{ Recall}}}}$$

Accuracy indicates ML classifiers correctness in the diagnosis of whether a patient is diabetic or non-diabetic using Eq. (10).

10 $${\text{Accuracy }} = \frac{{{\text{TP }} + {\text{ TN}}}}{{{\text{TP }} + {\text{ TN }} + {\text{ FN }} + {\text{ FP}}}}$$

MSE is a measure of the loss function using Eq. (11).

11 $${\mathrm{MSE}} = \frac{1}{{{\text{m *}}\mathop \sum \nolimits_{j = 1}^{m} \left( {X_{j} - \hat{X}_{j} } \right)}}$$

**Fig. 21:**
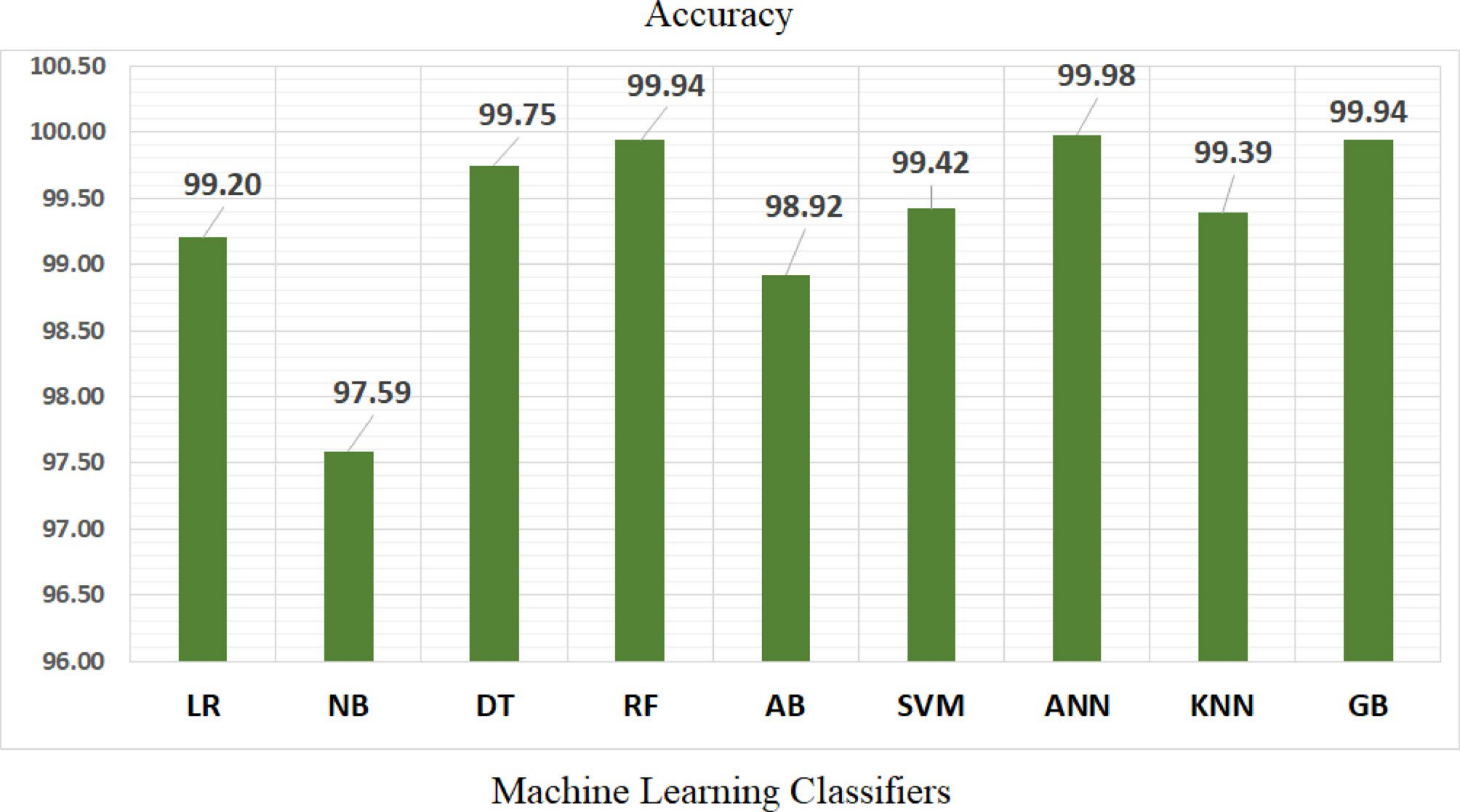
ML algorithms and accuracy.

Table 5 represents the efficiency comparisons of multi-class classification algorithms is evaluated based on five factors, including Training Score, MSE, R^2^ score, Precision, and AUC. The accuracy comparisons achieved by the nine classifiers is illustrated in Fig. 21.

**Table 5:** A comparison of ML techniques of training score, MSE, R2 score, precision, and AUC.

Model	Precision	Recall	F1 Score	AUC	MSE	R^2^ Score	Training score
NN	*0.9998*	*0.9998*	*0.9998*	*0.9998*	*0.0002*	*0.9998*	*1.000*
RF	0.9994	0.9994	0.9994	1.0000	0.0006	0.9996	1.0000
LR	0.9920	0.9920	0.9920	0.9994	0.0111	0.9911	0.9942
NB	0.9766	0.9759	0.9759	0.9989	0.0241	0.9807	0.9769
SVM	0.9942	0.9942	0.9942	0.9999	0.0058	0.9953	0.9967
AB	0.9895	0.9892	0.9892	0.9991	0.0108	0.9913	0.9901
KNN	0.9939	0.9939	0.9939	0.9989	0.0094	0.9925	0.9967
DT	0.9975	0.9975	0.9975	0.9988	0.0025	0.9980	0.9993
GB	0.9994	0.9994	0.9994	0.9999	0.0006	0.9996	1.0000

The ML techniques are implemented using Jupiter Notebook and the Python programming language. All previous studies have been performed on the PID dataset only for older ages of female’s gender. We need to extend the study to take into consideration the small ages of both genders, male/female, and add other diabetes dataset types that affect multiclassification to make a strong study. The new DTD dataset includes male/female patients with small and old ages to study effectively the diabetes types. In addition, the new input attributes are generated to support the decision-making of the multiclassification process. The aim of creating new features in the DTD dataset is essential to increase the accuracy and high predictive power of a model.

**Fig. 22:**
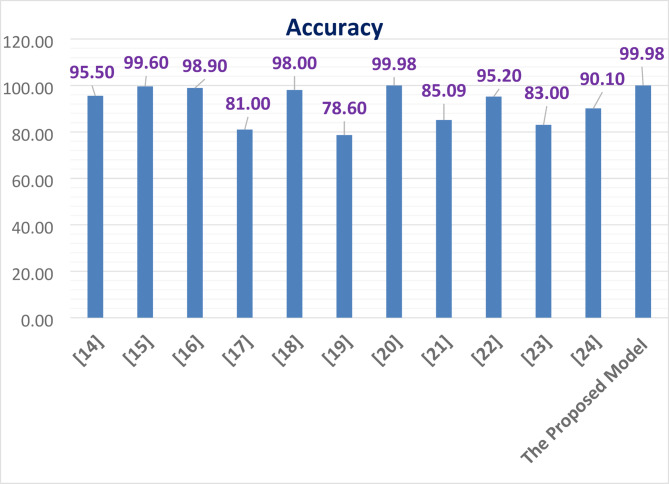
A comparison between the proposed model and other literature.

Table 6, Table 7 and Fig. 22 can conclude the supported model outperforms with high accuracy than other algorithms.

**Table 6:** A comparison of the ML models reviewed and the proposed model.

Study	Dataset	Preprocessing	Method	Summary
Reza MS, Amin R, Yasmin R, et al.^14^	PID, simulated dataset and local healthcare dataset	Normalization of input features. Merging multiple datasets with real data and simulated data. Handling class imbalance for effective model training	The study introduces two stacking-based ensemble models for classifying diabetes. It combines predictions from multiple ML and DNN models. Model evaluation employed a Train-test split and 5 fold cross-validation	Simulated dataset with NN obtained high accuracy of 95.50%. PID dataset obtained Train-test split accuracy of 75.03% and 5 fold CV accuracy of 77.10%. On combined datasets, using classical and DNN obtained an accuracy ranged from 92 to 95%
Hameed EM, Joshi H, Ismael AAA^15^	Combined dataset of PID and German Society Dataset, PID and German Society Dataset	Standard preprocessing include feature scaling, handling imbalanced classes	They used RF and GB classifiers to combine and compare performance across individual datasets and the merged dataset	PID dataset achieve accuracy of 0.817with RF. German Society Dataset achieve accuracy of 0.996 with GB and 0.994 with RF. In Combined Dataset: GB accuracy is 0.991 and RF accuracy is 0.988
Abnoosian K, Farnoosh R, Behzadi MH^16^	Structured dataset collected in Iraq	Clean duplicate sample, impute missing data, normalization, standardization, feature selection, and k-fold cross-validation	They used KNN, SVM, RF, AB, and NB for Multi-class classification of diabetes states	Demonstrated strong results in predicting multiple diabetes classes including prediabetes with high accuracy performance and area under the curve performance is 0.999
Tasin I, Nabil TU, Islam S, Khan R^17^	PID and Bangladesh (RTML)	Apply Feature Selection and apply SMOTE and ADASYN to handle imbalance class	They used DT, SVM, RF, L R, KNN and EGB binary classification with imbalance resolution. Use LIME and SHAP to understand the model prediction of results	Extreme Gradient Boosting with the ADASYN on class imbalance achieve accuracy of 81% and F1-score of 0.81 and AUC of 0.84
Jain A, Singhal A^18^	The diabetes dataset is PID and the food (HFD) Dataset	Applied SMOTE with Bat algorithm, nature-inspired optimization for feature selection and model tuning	They used ant colony optimization, Bat, Cuttlefish, Elephant Herd Optimization and Artificial Bee algorithms combined with voting ensemble techniques. They make hyperparameter tuning of ML models through the Hybrid Bat Algorithm, integrating optimization techniques with ensemble learning approaches	Both studies reported high accuracies. Highlighted the effectiveness of hybrid approaches using nature-inspired algorithms to optimize classifier performance
Salem Alzboon M, Alqaraleh M, Subhi Al-Batah M^19^	PID Dataset	Cleaning and standardization, while feature extraction and selection are performed using PCA in conjunction with RFE	They used LR, DT, RF, S VM, NB, GB, and NN classifiers and performed comparative performance evaluation of traditional ML classifiers	NN achieved the highest accuracy, followed by RF
Hasan M, Yasmin F^20^	Kaggle- real-world diabetes dataset	DNet, a Hybrid model that combines CNN and LSTM for Feature extraction and sequential learning Batch Normalization and Dropout are employed for robust regularization	They used LR, SVM, NB, RF, AB, GB, ET, XGB, and DL hybrid architecture for classification	Hybrid DNet model achieved high accuracy, area under the curve achieved 99.98%
sAhamed BS, Arya MS, Sangeetha SKB, Auxilia Osvin N V^21^	ADCU	Implemented using the JNN tool environment	The study used an ANN model to predict diabetes	The model showed high accuracy in predicting diabetes, demonstrating the potential of using ANN for early detection and healthcare applications
Ahamed BS, Arya MS, Sangeetha SKB, Auxilia Osvin N V^22^	PIMA	They used the R data manipulation tool, imputed the missing values with the mean value in the KNN algorithm, and they also used a feature selection technique to select the highly correlated data and dimension reduction to remove the outliers	The study compared different ML techniques (e.g., DT, SVM, KNN, RBF, ANN, and MDR) for diabetes prediction and type classification	DT and SVM achieved higher accuracy in predicting the type of diabetes
Krishnamoorthi R, Joshi S, Almarzouki HZ, et al.^23^	PIMA	They replaced the missing values with the mean value, then they used Pearson’s correlation technique and the WEKA analysis tool	They used ML techniques (e.g., DT, NN, KNN, RF, NB, AB, LR, and SVM) classifiers	Obtain high accuracy with regression models, making it a robust solution for diabetes prediction in healthcare
Afsaneh E, Sharifdini A, Ghazzaghi H, Ghobadi MZ^24^	PIMA dataset	They replaced the missing values with the Mean value	They used ML and DL models (e.g., ANN, CNN, LR, KNN, SVM, RF, and DL. They applied the Scikit learning Toolkit and the grid search algorithm with fine-tuned hyperparameters	The CNN and ANN algorithms exhibited higher accuracy in automated diagnosis and prediction diabetes
The Proposed Model	DTD	We replaced the missing values using the MICE and smote techniques and fine-tuned the hyperparameters using the ANN Algorithm with PSO	We use ML multiclassification techniques like LR, NB, DT, RF, AB, KNN, GB, and ANN with the PSO technique	ANN shows higher accuracy with the diabetes multiclassification dataset

**Table 7:** A notations of Table 6 and their descriptions.

Notation	Description
Simulated Dataset	The information was collected from the Pabna Diabetes Hospital, Pabna, Bangladesh
German Society Dataset	The dataset was taken from the hospital in Frankfurt, Germany. It has 2000 records and 9 features. Hence, the combined dataset includes 2,768 records and 9 features. Every patient inthesedatasetswasolderthan21andfemale
Iraq Dataset	The Iraqi Patient Dataset for Diabetes (IPDD)^29^ was obtained from 1000 samples, including 565 males and 435 females aged 20–79 years old, during in-hospital physical examinations at the Specialized Centre for Endocrinology and Diabetes
RTML Dataset	The dataset is private from Rownak Textile Mills Ltd, Dhaka, Bangladesh. The dataset comprises six features, that is, pregnancy, glucose, blood pressure, skin thickness, BMI, age, and outcome of diabetes from 203 female individuals aged between 18 and 77
HFD Dataset	contains similar information of the PID dataset about patients’ symptoms, including pregnancy occurrence (number of times), blood sugar, insulin levels, and Skin Thickness, as well as their height is to weight ratio, i.e., Body Mass Index, age, family history of diabetes, and whether health is prone to get diabetes in the future
ADCU	The dataset was gathered from the documentation of the Association of the diabetic city of Urmia, which contains 1004 samples with 9 attributes such as Pregnancies, Plasma glucose concentration, Diastolic Blood Pressure, Triceps Skin Fold Thickness, Body Mass Index, Diabetes Pedigree Function, Age, and Diabetes
PIMA	Pima Indian Diabetes Dataset (the dataset is available in the UCI Repository owned by the National Institute of Diabetes and Digestive and Kidney Diseases. The dataset consists of 9 attributes: pregnancy, glucose, blood pressure, insulin, skin thickness, BMI, diabetes pedigree function, age, and outcome. The total number of instances are768. The PIMA dataset is used for the prediction of diabetes.)
JNN	Just Neural Network
EGB	Extreme Gradient Boosting
ADASYN	Adaptive Synthetic Sampling
PCA	Principal Component Analysis
RFE	Recursive Feature Elimination
ET	Extra Trees
LSTM	Long Short-Term Memory network
LR	Logistic Regression
KNN	K Nearest Neighbours
SVM	Support Vector Machine
RF	Random Forest
GB	Gradient Boosting
XGB	XG Boost
DT	Decision Tree
NB	Naive Bayes
AB	Ada boost
RBF	Radial Basis Function
ANN	Artificial Neural Network
MDR	Multifactor Dimensionality Reduction
PSO	Particle Swarm Optimization

## Conclusions and future work

Early detection of diabetes poses a significant challenge within healthcare systems, particularly due to the complex nature of the disease and its varying manifestations. This study demonstrates the powerful potential of supervised ML techniques in accurately detecting and classifying multiple types of diabetes. By leveraging a multiclass classification framework trained on the integrated DTD dataset, which combines patient records from Pediatrics, PID, Pone, and Gestational Diabetes sources, the developed ML system effectively classifies Type 1, Type 2, Gestational, and Normal (non-diabetic) cases. A robust preprocessing pipeline, including MICE-based imputation and SMOTE for class balancing, ensured data quality and fairness. Multiple ML algorithms, such as LR, NB, DT, RF, AB, GB, SVM, KNN, and ANN, were evaluated, with model tuning performed using PSO to optimize hyperparameters. The system’s transparency was enhanced through SHAP analysis and detailed performance visualizations (Confusion Matrix, ROC Curves, and Learning Curves), offering clinical interpretability and confirming the model’s reliability. Notably, classifiers like ANN achieved superior performance, recording up to 99.98% accuracy with minimal misclassification. These models outperformed other techniques reported in the literature, including in external validation tasks using two diverse diabetes datasets: one with 100,000 records and another containing 12 distinct diabetes types across 34 features. The system’s ability to generalize effectively across both simple and complex datasets underscores its adaptability, robustness, and real-world clinical applicability. The integration of this ML system into clinical workflows and self-management protocols has the potential to revolutionize diabetes care, empowering healthcare professionals and patients alike with timely, data-driven insights. By accurately identifying indicators such as insulin levels for Type 1 and Type 2 diabetes and POGTT results for Gestational diabetes, the system facilitates early diagnosis and supports proactive disease management. The comprehensive evaluation using multiple performance metrics, including Precision, Accuracy, MSE, R^2^ Score, and AUC confirms the adequacy and reliability of the developed system, establishing it as a powerful tool for automated, multi-type diabetes diagnosis. Future extensions of this work could include automating diabetes prediction, developing an Android application for diabetes monitoring, integrating genetic algorithms with prediction mechanisms, and exploring enhancements by incorporating medical scan images such as those related to eye and skin diseases. The designed system, equipped with ML multiclassification algorithms, holds promise for predicting and diagnosing various diseases beyond diabetes.

## Supplementary Information

Below is the link to the electronic supplementary material.

Supplementary Material 1

## Data Availability

The **DTD** dataset is not publicly shared due to the imperative need for patient data security. Access to this dataset is strictly limited to authorized individuals who have obtained a clear written agreement from the hospital manager. The data supporting the findings of this study are accessible through the hospital repository system, but only to those who have authorized access. However, it is important to note that restrictions apply to the availability of these data, as they were used under license for the current study and are therefore not publicly available. Should you require access to the data, please submit a reasonable request to the corresponding author. However, please be aware that permission from both the hospital manager and the head of the Endocrinology/Diabetes department at Mansoura University Children Hospital is required. The PIMA dataset is originally from the National Institute of Diabetes and Digestive and Kidney Diseases 31. The PIMA is available at: [https://www.kaggle.com/datasets/uciml/pima-indians-diabetes-database]^34^. The Pone dataset Participants were recruited from the Freedom from Diabetes Clinic in Pune, India. Of the 7839 T2D patients [32]. The Pone is available at: [https://pmc.ncbi.nlm.nih.gov/articles/PMC11068193/#sec015]. The Gestational dataset is collected from Government and private Multispecialty Hospitals in Thanjavur district, Tamil Nadu, India [33]. The Gestational is available at: [https://www.kaggle.com/code/medahmedkrichen/gestational-diabetes/input]. The Pediatrics dataset is obtained from the Mansoura University Children’s Hospital repository system, Medicine Faculty, and Dakahlia Governorate of Egypt^30^. The first external dataset (diabetes_prediction_dataset) is available at: [https://www.kaggle.com/datasets/dat00700/diabetes-prediction-dataset]. The second external dataset (diabetes_dataset) is available at: [https://www.kaggle.com/datasets/ankitbatra1210/diabetes-dataset].

## Code availability

The codes repository for this work is supplied here: GitHub—abeereg/Diabetes.
