# Supplementary material for: Pediatric diabetes prediction using machine learning
Source: Sci Rep. 2026 Jan 15;16:1979. doi: 10.1038/s41598-025-24964-y (PMC12808089; doi:10.1038/s41598-025-24964-y)
Supplement: Supplementary file 1 — Supplementary Material 1 [file 41598_2025_24964_MOESM1_ESM.docx]

**Appendix A: The DTD Dataset Collection is a unified dataset compiled from four different diabetes datasets according to the following steps:**

**Step 1: Understand four diabetes Datasets using:**

**PIMA Diabetes Dataset**

- **Attributes:** Pregnancies, Glucose, BloodPressure, SkinThickness, Insulin, BMI, DiabetesPedigreeFunction, and Age.
- **Target:** Outcome (diabetic or not diabetic).

**Pone Diabetes Dataset**

- **Attributes:** Patients_S No., Consentacceptedyn, ReportInterpretation, GTT_FBSL, GTT_PP2, Duration_of_Diabete, On_Insulin_yn, On_Diabetes_Medicine_yn, Heightcms, Weightkgfirstoccurance, Weightkglastoccurance, BMIfirstoccurance, BMIlastoccurance, HbA1Cfirstoccurance, HbA1Clastoccurance, FastingBloodGlucosemgdlfirstoccurance, FastingBloodGlucosemgdllastoccurance, InsulinFastinguUmlfirstoccurance, InsulinFastinguUmllastoccurance, HOMA_IR_baseline, HOMA_IR_endline, HOMA_Beta_baseline, and HOMA_Beta_endline.
- **Target:** Type 2 diabetes.

**Gestational Diabetes Dataset**

- **Attributes:** Age, No of Pregnancy, Gestation in previous Pregnancy, BMI, HDL, Family History, unexplained prenetal loss, Large Child or Birth Default, PCOS, Sys BP, Dia BP, OGTT, Hemoglobin, Sedentary Lifestyle, Prediabetes
- **Target:** binary classification (Gestational diabetes or non-Gestational diabetes).

**Pediatrics Diabetes Dataset**

- **Attributes:** Age, Duration, BloodPressure, Cholesterol, Creatinine, Acetone, HbA1c, Insulin, PCPeptide, FCPeptide, PBGlucose, FBGlucose, RBGlucose, PH, HCO3, Na, and K.
- **Target:** Diagnosis binary classification (diabetic or not diabetic).

**diabetes_prediction_dataset External Dataset**

- **Attributes:** gender, age, hypertension, heart_disease, smoking_history, bmi, HbA1c_level, and blood_glucose_level.

**Target:** diabetes

**Step 2: Align the Column Names**

**In the DTD dataset, we align the column names as follows:**

Identify overlapping features and align the column names, rename columns into new consistent names across the unified dataset, and map similar column attributes, e.g.:

"**Age**" in pone, PIMA, Pediatrics, and Gestational Datasets is converted to **→** "**Age**" in the DTD dataset.

"**Pregnancies**" in the PIMA dataset, and "**No of Pregnancy**" in the Gestational dataset are converted to **→** "**NPregnancies**" in the DTD dataset. "**HbA1Cfirstoccurance**" in Pone dataset, and "**HbA1c**" in Pediatrics Dataset are converted to **→** "**HbA1c**" in DTD dataset.

"**BMIfirstoccurance**" in the pone dataset, “**BMI**" in PIMA, Pediatrics, and Gestational are converted to **→** "**BMI**" in the DTD Dataset.

"**GTT_PP2**" in the pone dataset, "**POGTT**" in the pediatrics dataset, and "**OGTT**" Gestational Dataset are converted to **→** "**POGTT**" in the DTD dataset.

" **GTT_FBSL** " in the pone dataset and "**FOGTT**" in the pediatrics dataset are converted to **→** "**FOGTT**" in the DTD dataset.

"**Glucose**" in the PIMA dataset, and "**PBGlucose**" in the Pediatrics Dataset are converted to **→** "**PGlucose**" in the DTD dataset. "**FastingBloodGlucosemgdlfirstoccurance**" in the Pone dataset, and "**FBGlucose**" in the Pediatrics Dataset are converted to **→** "**FGlucose**" in the DTD dataset.

"**Insulin**" in the PIMA and Pediatrics dataset, "**InsulinFastinguUmlfirstoccurance**" in the Pone the dataset are converted to **→** "**Insulin**" in the DTD dataset.

"**BloodPressure**" in PIMA Dataset, "**Dia BP**" in Gestational Datasets, and "**BloodPressure**" in Pediatrics Dataset are converted to **→** "**BPressure**" in the DTD dataset.

"**Outcome**" in the PIMA dataset, "**Class Label (GDM /Non GDM)**" in the Gestational Dataset, "**Diagnosis**" in the Pediatrics Dataset, and “**diabetes**” in the diabetes_prediction_dataset are converted to **→"Diagnosis**" in the DTD Dataset.

**In the diabetes_prediction_dataset External Dataset, we align the column names as follows:**

“**age**” in diabetes_prediction_dataset is converted to **→** "**Age**" in the DTD dataset.

“**gender**” in diabetes_prediction_dataset is converted to **→** "**Sex**" in the DTD dataset.

“**HbA1c_level”** in diabetes_prediction_dataset is converted to **→** "**HbA1c**" in the DTD dataset.

“**bmi**” in diabetes_prediction_dataset is converted to **→** "**BMI**" in the new DTD Dataset.

“**blood_glucose_level**” in diabetes_prediction_dataset is converted to **→** "**PGlucose**" in the DTD dataset.

“**heart_disease**” in diabetes_prediction_dataset is converted to **→** "**BPressure**" in the DTD dataset.

“**diabetes**” in diabetes_prediction_dataset is converted to **→"Diagnosis**" in the DTD Dataset.

**Step 3: Standardize Units**

Ensure all features are measured in the same units (e.g., glucose levels in mg/dL or mmol/L) as indicated in table 1.

**Step 4: Handle Missing Features**

For features missing in all datasets with NaN values, replace them with the MICE imputer.

***Step 5: Combine and Save the Datasets***

Concatenate the datasets into a single DTD.csv file.

**Appendix B: ANN Model**

This workflow showcases a combination of ANN for multi-class classification of the DTD dataset[35]:

***Input Layer***

The first layer accepts 12 input features using Eqs. (12):

| X_12_=[X_1_, X_2_, X_3_, ...., X_12_] | (12) |
| --- | --- |

- Initialize the weight parameters ($\theta)$, random real numbers between 0 and 1, using Eqs. (13):

| $\theta$ = [$\theta$_0_, $\theta$_1_, ..., $\theta$_12_] | (13) |
| --- | --- |

- Let the bias node be (b). Let a counter (j) be used to track the input features and n is the total number of input features.
- **Linear Transfer Function**
  For feedforward propagation, calculate (a_j_)​ using Eqs. (14):

| $a_{j}$= $\sum_{j=1}^{j=n} {\theta_{j}^{T}X}_{j}+b$ | (14) |
| --- | --- |

Where $a_{j}$ is a Linear combination of inputs and weights for the j-th feature.

- Replace the bias term (b) with $\theta_{0}X_{0}$ where $X_{0}=1 using Eqs. (15):$

| $a_{j}$=${\theta_{0}+\theta_{1}X}_{1}+{\theta_{2}X}_{2}+\ldots+{\theta_{12}X}_{12}$ | (15) |
| --- | --- |

**Hidden Layer**

The second dense layer is the hidden layer, which is another fully connected layer. It uses the Rectified Linear Unit (ReLU) activation function to learn complex patterns. The ReLU activation function helps avoid the vanishing gradient problem and enables the network to learn complex mappings. The model is compiled using a swarm optimization technique to fine-tune hyperparameters like optimizer and adapts the learning rate during the training process [36].

- **ReLU Activation Function**
  Apply the ReLU activation function to calculate the predicted output: ($a_{(j)}^{(i)}$) using Eq. (16) and Figure A1. The predicted output ($a_{j}^{i}$) of a neuron is sent as input to all neurons in the next layer hidden layer.

|  |  |
| --- | --- |
| \| $a_{j}=max(0, a)$= $\frac{a+\vert a\vert}{2}$=$\left\{ \begin{aligned} a if a>0, \\ 0 otherwize \end{aligned} \right.\left\{ \begin{aligned} 1 if a>0, \\ 0 if a<0 \end{aligned} \right.$ \| (16) \| \| --- \| --- \|   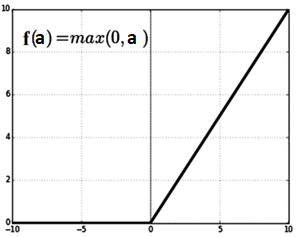 | |

**Figure A1.** Relu Function

ReLU ensures non-linearity, reducing vanishing/exploding gradient problems [37].

- **Cross-Entropy Loss Function**
  Minimize the cross-entropy loss J(θ) using Eq. (17):

| $J\left( \theta\right)=-\log L\left( \theta\right)$ | (17) |
| --- | --- |

Where L $(\theta$)$=-\sum_{j=1}^{n} (\prod_{j=1}^{n} ={P(y_{j}=y}_{j}^{n})|X_{j}^{n};\theta_{j}^{n}$

| The derivative is given by Eq. (18):   \| $J\left( \theta\right)=-\log L\left( \theta\right)$ \| (18) \| \| --- \| --- \|   Where L $(\theta$)$=-\sum_{j=1}^{n} (\prod_{j=1}^{n} ={P(y_{j}=y}_{j}^{n})\vert X_{j}^{n};\theta_{j}^{n}$   - **Derivative of the Loss Function**  \| The derivative is given by Eq. (19):   \| Y$=-\sum_{j=1}^{n} (\prod\left\{ y^{\left( n \right)}=j \right\}-$ $F_{j}$(${X_{j}}^{\left( n \right)}$;$\theta_{j})$) ${X_{j}}^{\left( n \right)}$ \| (19) \| \| --- \| --- \| \|  \| \| --- \| --- \| --- \| --- \|  - **Gradient Descent Updates**   For the bias term θ_0_​, use Eq. (20): | |  | |  |
| --- | --- | --- | --- | --- | --- | --- | --- | --- | --- | --- |
| $\theta_{0}:=\theta_{0}-\alpha*\frac{1}{n}*\sum_{j=1}^{n} ({a_{j}}^{\left( i \right)})-{Y_{j}}^{\left( i \right)})X_{0}^{\left( i \right)})$ | *(20)* | |  |  |
| Update the weights θj​ using Eq. (21): | | |  | |
| $\theta_{j}:={{\theta_{j}}^{\left( i \right)}}-\alpha*\left⟦ (\frac{1}{n}*\sum_{j=1}^{n} ({a_{j}}^{\left( i \right)})-{Y_{j}}^{\left( i \right)})X_{j}^{\left( i \right)})+\frac{\lambda}{n}{{\theta_{j}}^{\left( i \right)}} \right⟧$ | | | *(21)* | |
| Where $\alpha$ is the Learning rate, $\lambda$ is the Regularization parameter, $Y_{j}$true label for the i-th sample and j-th feature, and $X_{j}^{\left( i \right)}$Input feature values for the j-th feature in the i-th sample. | | |  | |

- **Error Backpropagation**
  Compute the error for the node *using Eq. (22):*

| $\delta_{j}^{l}=a_{j}^{i}-y_{j}$ | (22) |
| --- | --- |

- For hidden layers:

Calculate error ($\delta_{j}^{l})$ Delta in the hidden layers *using Eq. (23):*

| $\delta_{j}^{l}=\left( {{\theta_{j}}^{\left( i \right)})}^{T} \delta_{j}^{l+1} \right)$⊙*g′*$(Z^{\left( i \right)})$ | (23) |
| --- | --- |

- Compute Cumulative Gradient (Delta)$\Delta_{ij}^{l}$. The Cumulative Sum of Delta for all layers of the training set *using Eq. (24):*

| $\Delta_{ij}^{l}:=\Delta_{ij}^{l}+\delta_{j}^{l+1} {(a_{j}^{i})}^{T}$ | (24) |
| --- | --- |

- Compute the regularized cost Function using Eqs. (25-26).

| $D_{i,j}^{\left( i \right)}:=\frac{1}{n}\left( \Delta_{i,j}^{i}+\lambda\theta_{i,j}^{\left( i \right)} \right) ,for j\neq0$ | (25) |
| --- | --- |
| $D_{i,j}^{\left( i \right)}:=\frac{1}{n}\left( \Delta_{i,j}^{i} \right) , for j=0$ | (26) |

***Output layer***

The model is designed to classify into four categories: Normal, Type 1 Diabetes, Type 2 Diabetes, and Gestational Diabetes. The output layer receives the processed values from the previous layer (usually the last hidden layer). These values are passed through a set of weights and biases corresponding to the four classes. Then the output layer will have four neurons. Each neuron computes a weighted sum of the inputs and applies the SoftMax activation function.

- **SoftMax Function**

To normalize outputs into probabilities as indicated in Figure A2, compute $a_{j}^{i}$ using Eq. (27).


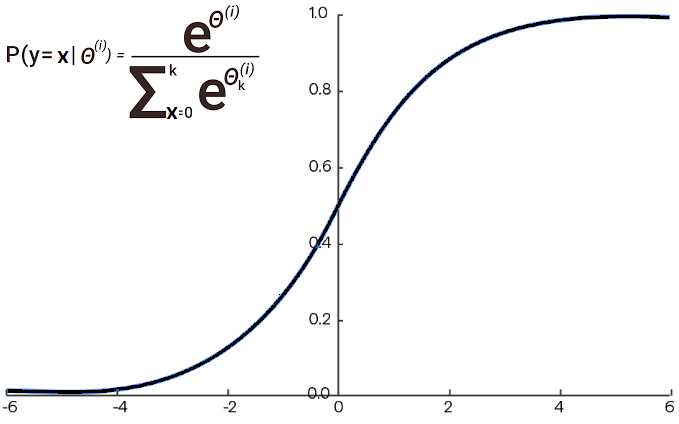


**Figure A2.** SoftMax Function

| P(y=$k\vert X_{j};\theta_{j}^{(i)})$= $\frac{{{exp}^{\theta}}_{j}^{T}X_{j}}{\sum_{j=1}^{n} {{exp}^{\theta}}_{j}^{T}X_{j}}$ , $k\in\left\{ 0,1,2,3 \right\} and j\in\{1,2, \ldots, 12\}$ | (27) |
| --- | --- |

**Appendix C: Confusion Matrix and Multiclass ROC Classification for the Machine learning Techniques**

| **Terminology** | **Description** |
| --- | --- |
| **Confusion Matrix (CM)** | Shows the performance of a classification model. |
| **True Positive (TP)** | Correctly predicted instances of a class |
| **False Positive (FP)** | Instances misclassified as a given class (wrongly predicted positives) |
| **False Negative (FN)** | Instances of a class that were not predicted correctly (missed positives) |
| **True Negative (TN)** | Instances correctly identified as not belonging to a specific class |
| **True Positive Rate (TPR)** | A metric used to evaluate the performance of a classification model, especially in binary or multiclass classification tasks, is “the proportion of actual positives that are correctly identified by the model”. |
| **False Positive Rate (FPR)** | a key performance metric in classification problems, especially when you care about how many negative cases were incorrectly flagged as positive, “the proportion of actual negatives that were incorrectly classified as positives”. |
| **ROC curve** | shows the performance of a multiclass classification model for four diabetes types compared against the rest. |
| **Area Under the Curve (AUC)** | The area under the ROC curve, evaluating the model’s ability to distinguish between classes (closer to 1 = better) |
| **X-axis** | represents the False Positive Rate (FPR): the proportion of negatives incorrectly classified as positives |
| **Y-axis** | The y represents the True Positive Rate (TPR): the proportion of positives correctly classified |

Table A1 Main Terminology of Confusion Matrix and ROC Curve

The DTD sample of 12 features and DiagnosisType output attribute are shown in Figure A3.


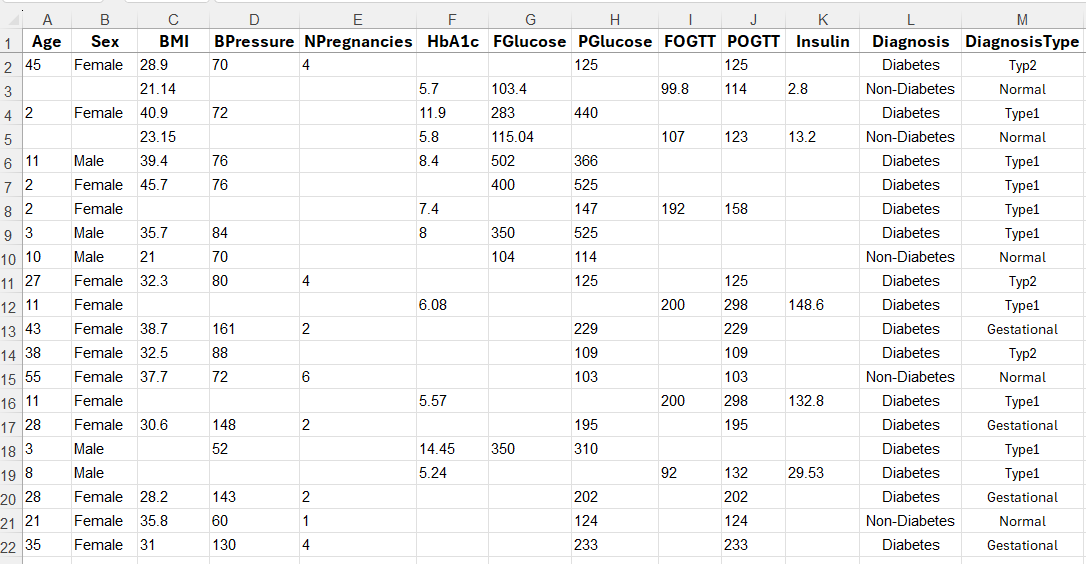


**Figure A3.** A Sample of DTD Dataset

Here is a table representation of a confusion matrix of the ML techniques, with each cell labelled TP, FP, FN, and TN.

| **Actual\Predicted** | **Normal** | **Type 1** | **Type 2** | **Gestational** |
| --- | --- | --- | --- | --- |
| **Normal** | **TP** | FN | FN | FN |
| **Type 1** | FP | **TP** | FN | FN |
| **Type 2** | FP | FP | **TP** | FN |
| **Gestational** | FP | FP | FP | **TP** |

Table A2. Confusion Matrix with the corresponding TP, FP, FN and TN of Diabetes Types

Table A1 describes the main terminology of the confusion matrix, and Table A2. Indicate the confusion matrix with the corresponding TP, FP, FN, and TN cells. This confusion matrix shows the classification performance of a model on a resampled dataset across four classes: Normal, Type1, Type2, and Gestational. Each cell indicates the number of instances of actual (rows) versus predicted (columns) class labels.

After applying resampling to address the original class imbalance in the diagnosis dataset, all models showed improved generalization on a balanced test set with nearly uniform class distribution. NN achieved the highest accuracy of 99.98, followed by GB and the unidentified RF model, with near-perfect accuracy (0.9994), F1-score (0.9994), AUC (1.0000), and minimal mean squared error (0.0006), while maintaining perfect or near-perfect training scores, suggesting highly effective learning without overfitting. LR and SVM also performed excellently, especially after PSO optimization, though their improvements were more incremental compared to complex ensemble models. NB, while showing respectable results (accuracy: 0.9759), it struggled slightly with class 2, reflecting limitations in modelling complex distributions. AB, KNN, and DT delivered robust accuracy (above 0.98), and PSO consistently boosted performance across all classifiers. The use of PSO proved particularly beneficial for hyperparameter tuning, slightly enhancing already strong metrics, especially in non-linear or kernel-based models.

**Here is detailed information for Figure 12, which displays the Confusion Matrix, ROC Curve, and Learning Curve for the RF models:**

The confusion matrix (CM) for the RF model predicts four classes of diabetes types: Normal, Type 1, Type 2, and Gestational. The CM compares actual outputs with predicted outputs using the MUCHD dataset. Diagonal values contain the corrected predictions. Normal: 3004. Type 1: 3004. Type 2: 3003. Gestational: 3003. Out-diagonal values provide misclassifications of patients: Type 2 is misclassified as Gestational once and vice versa. All other diabetes types are presented correctly. The ROC curve for the RF classifier overlaps and reaches the top-left corner of the plot, indicating the FPR is near 0 and the TPR is 1.0, which clarifies near-perfect classification performance. This matches with an AUC close to 1.0 for all classes. The model significantly outperforms; the dashed diagonal line represents random guessing. AUC is 0.5 with an illustrated distance between the line and the ROC curves. The learning curve illustrates how accuracy varies with increasing training size for both the training and validation sets. The training accuracy remains perfect at 1.0 across all training data sizes. The validation accuracy initially was slightly low but improved gradually as the training size increased, reaching nearly 99.96% with the largest dataset. This means the RF model has minimal overfitting and high generalization performance.

**Here is detailed information for Figure 13, which displays the Confusion Matrix, ROC Curve, and Learning Curve for the KNN models:**

The CM for the KNN model indicates the actual Normal cases are 3004, while 2993 were correctly classified, with eleven misclassified patients. Similarly, Type 1 and Type 2 classes had 2983 and 2987 correct predictions out of 3004 each, showing very low misclassification rates. The Gestational class achieved near-perfect performance with 3003 out of 3004 correctly predicted. The multiclass ROC curve for the KNN model shows the tight clustering of the ROC curves at the top-left implies high sensitivity and specificity for all categories. The learning curve for the KNN model shows exceptionally high accuracy for both training and validation sets across all training sizes. From the very beginning, the model achieves over 98% accuracy, and as the training size increases, both curves continue to rise near 99.6% (training) and 99.5% (validation).

**Here is detailed information for Figure 14, which displays the Confusion Matrix, ROC Curve, and Learning Curve for the DT models:**

The CM for the DT model achieved perfect classification for the Normal and Type 1 classes, correctly identifying all 3004 samples in each category. For the Type 2 class, 3000 samples were correctly classified, with only 4 misclassified as Gestational. Similarly, the Gestational class presents 2993 correct predictions, with eleven misclassified patients as Type 2. The ROC curve for the DT model indicates the model is highly effective, with minimum confusion between types. The high AUC values support the model’s excellent sensitivity and specificity. The learning curve for the DT model presents high accuracy performance for both training and validation datasets. The training accuracy is close to 1.0 across all training data sizes. The validation accuracy fluctuates between 99.5 to 99.8. Both curves preserve high values with low variation at increased the training data size increases. This led to model effectiveness in generalizing well to unknown data.

**Here is detailed information for Figure 15, which displays the Confusion Matrix, ROC Curve, and Learning Curve for the GB models:**

The CM for the GB model shows that the model correctly classifies almost every data sample for each type, with the minimum number. 3004 Normal and Type 1 instances are correctly predicted, while two Type 2 samples are incorrectly labelled as Gestational, and all Gestational samples are correctly classified. The ROC curve for the model illustrates the overlap of curves near the upper boundary. The learning curve indicates robust performance. The training accuracy perfectly remains constant at 1.0 for all data samples. The validation accuracy consistently increases with the training sample size. This suggests the model generalizes and has minimum variance without overfitting.

**Here is detailed information for Figure 16, which displays the Confusion Matrix, ROC Curve, and Learning Curve for the NB models:**

The CM for the NB model performs well for the Normal class, with all 3004 samples. Type 1 has 53 samples incorrectly classified as Type 2, and Type 2 shows the most significant confusion, with 201 instances misclassified as Gestational, 17 as Type 1, 9 Gestational samples are misclassified as Type 2, and 1 as Normal. This indicates the model perfectly classifies Normal cases, but it has difficulty differentiating between Gestational, Type 2, and Type 1, especially between Type 2 and Gestational, showing overlapping feature distributions. This ROC curve illustrates that all classes are very close to the top-left corner, indicating a high TPR (sensitivity) and a low FPR, which leads to a strong performance with a very high AUC close to 1.0. The NB model learning curve, both curves show some fluctuation, with accuracy decreasing slightly with increased data instance sizes. This means the model may not generalize well with a low training data size. But with the largest training sizes, both curves converge at around 97.2% accuracy, indicating strong generalization and consistent performance.

**Here is detailed information for Figure 17, which displays the Confusion Matrix, ROC Curve, and Learning Curve for the AB models:**

The CM for the AB model presents that the Normal class is correctly classified for all 3,004 cases. Type 1 misclassifies 1 as Gestational and 6 cases as Type 2. Type 2 has 112 cases predicted as Gestational, while Gestational has 1 as Normal and 2 as Type 2. All ROC curves are clustered approaches the top-left corner of the curve, indicating a high TPR and low FPR. The learning curve illustrates an improvement in both training and validation accuracy with increased training samples and low performance with a small data size.

**Here is detailed information for Figure 18, which displays the Confusion Matrix, ROC Curve, and Learning Curve for the LR models:**

Using the CM for the LR model, the diagonal values illustrate the corrected classifications: 3003 of Normal cases, 2986 of Type 1, 2954 of Type 2, and 2995 of Gestational are correctly predicted. 1 of Normal case is misclassified as Gestational. 13 of Type 1 cases are misclassified as Type 2, and 5 are as Gestational. 11 of Type 2 cases are misclassified as Type 1, and 39 are as Gestational. 1 of the Gestational cases is misclassified as Normal, and 8 as Type 2. The model faces minor challenges when distinguishing between Gestational and Type 2, and to a minor extent between Type 2 and Type 1. The ROC curve uses a one-vs-rest strategy for 4 classes: Normal, Type 1, Type 2, and Gestational. Each curve shows the trade-off between the TPR and the FPR for one class versus the others. All curves are positioned close to the top-left corner, providing high sensitivity (recall) and low FPR across all classes. This led to a distinction between classes, with AUC values very close to 1.0. The learning curve indicates how accuracy advances with increasing training data size for both the training and validation datasets. A slight overfitting results in a small gap between training and validation accuracy, and it narrows as the training size exceeds around 99. 36.

**Here is detailed information for Figure 19, which displays the Confusion Matrix, ROC Curve, and Learning Curve for the ANN models:**

The CM for the ANN model presents two Normal cases that are incorrectly predicted as Type 1. All other predictions are perfectly accurate, with no confusion between Type 2, Type 1, and Gestational types. This achieves an overall accuracy of 99.98%. This is stable with an AUC close to 1.0 for all classes. The dashed diagonal line represents an AUC equal to 0.5, and the distance between this line and the ROC curves confirms that the model significantly outperforms random chance. The learning curve illustrates that the training accuracy remains consistently perfect at 1.0 across all training data sizes, indicating that the model fits the training data extremely well. The validation accuracy starts slightly lower but improves steadily as the training size increases, reaching approximately 99. 96 with the largest dataset. The very small gap between training and validation accuracy suggests that the model has high generalization performance with minimal overfitting. This plot indicates the model is robust, benefits from large data, and maintains superior predictive performance across various training set sizes.

**Here is detailed information for Figure 20, which displays the Confusion Matrix, ROC Curve, and Learning Curve for the SVM models:**

The CM of the SVM model shows high accuracy, with most predictions falling along the diagonal of correct classifications. Specifically, it correctly classified 3001 out of 3004 Normal cases, 2997 out of 3004 Type 1 cases, 2971 out of 3004 Type 2 cases, and 2998 out of 3004 Gestational cases. Most misclassifications occurred between Type 2 and Gestational, where 30 Type 2 cases were predicted as Gestational and 5 Gestational cases were predicted as Type 2. Minor confusion is also observed in a few Type 1 cases predicted as Type 2 or Gestational. The minimal misclassification between Normal and the other types suggests strong separation for that class, while some overlap remains between the diabetic subtypes. The ROC curve illustrates the multiclass classification performance of the SVM model; each curve represents how well the model distinguishes one class from the rest by plotting the TPR against the FPR. All curves are tightly clustered near the top-left corner of the graph, which indicates a high sensitivity and low FPR for all classes. This shape suggests that the SVM model has an excellent discriminative ability, with AUC values likely very close to 1.0. The curves explain that the model makes very few errors in differentiating between the types. The learning curve for the model demonstrates that as the training size increases, both training and validation accuracy steadily improve, with the validation curve closely tracking the training curve. This convergence indicates that the model is generalizing well and not suffering from overfitting or underfitting. The small gap between the two curves also reflects the model’s stability and robustness across different training sizes, making the SVM a highly reliable classifier.

Appendix D:

We use two external datasets in this study. The diabetes_prediction_dataset is utilized for prediction tasks, while the diabetes_Dataset is employed to evaluate the overall generalization ability of the system.

**All experiments were conducted using the diabetes_Dataset instead of the DTD dataset as follows:**

The system starts by loading a diabetes dataset and separating features from the target variable. It handles missing values using iterative imputation and standardizes the features for consistency. To address class imbalance, it applies by SMOTE is applied, resulting in a balanced dataset. A classifier is then trained on the resampled data, and its performance is evaluated using accuracy, classification reports, and ROC-AUC metrics. SHAP is used to interpret feature importance and visualize model explanations, as shown in Figure A4. A confusion matrix is generated to evaluate prediction accuracy across all classes. The system plots a multiclass ROC curve and a learning curve to visualize model performance and training behaviour. PSO is applied to optimize hyperparameters like the number of trees and depth in the RF model, and performance before and after tuning is compared. Finally, additional evaluation metrics such as accuracy are reported to provide a comprehensive assessment of the model's effectiveness.

The original class distribution was imbalanced across all categories (MODY: 5553, Secondary: 5479, Cystic Fibrosis-Related: 5464, Type 1: 5446, Neonatal Mellitus: 5408, Wolcott-Rallison Syndrome: 5400, Type 2: 5397, Prediabetic: 5376, Gestational: 5344, Type 3c: 5320, Wolfram Syndrome: 5315, Steroid-Induced: 5275, LADA: 5223). To address this imbalance, the dataset was resampled using the SMOTE technique, resulting in a uniform distribution of 5555 samples for each class.

Among the five evaluated models, such that RF, DT, NB, GB, and LR. GB model (after PSO optimization) demonstrated the best overall performance with an accuracy of 0.9028, F1-score of 0.9068, and AUC of 0.9962, while maintaining a strong training score of 0.917, indicating both high predictive accuracy and generalization capability as shown in Table A3. PSO-based hyperparameters tuning consistently improved metrics across most models, though gains were marginal in simpler models like Model (likely LR), which showed limited capacity to handle the dataset’s complexity. All models benefited from resampling, which equalized the class distributions, helping ensure fair evaluation across all 13 classes.


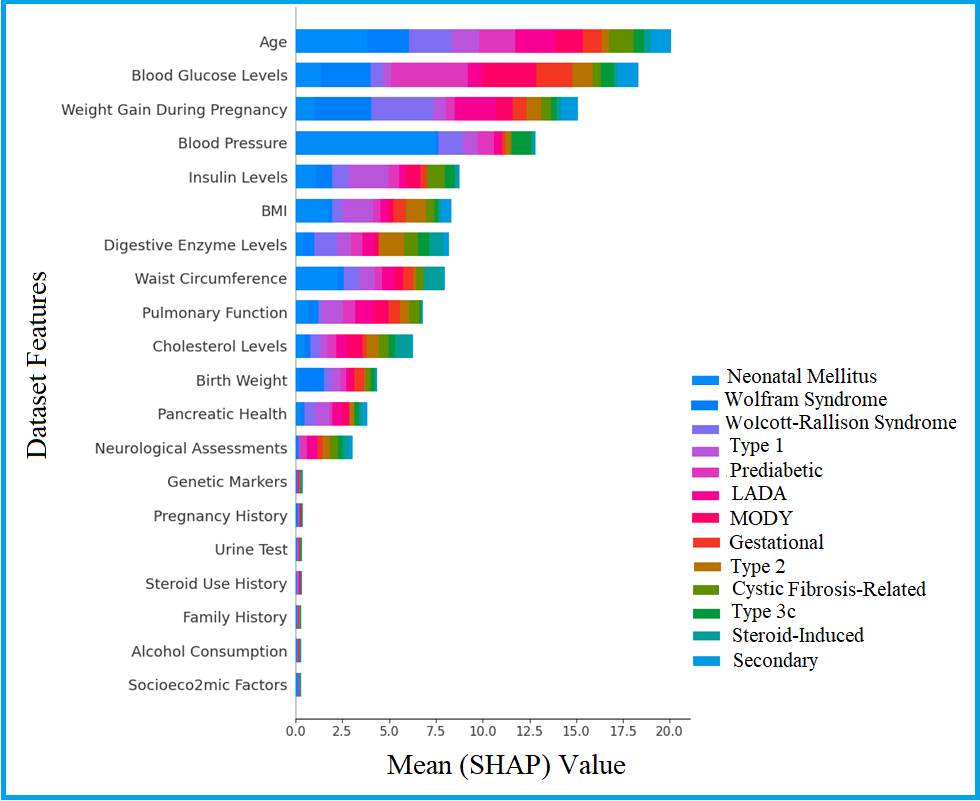


**Figure A4.** The SHAP Graph of diabetes_Dataset Dataset

The relationship between diabetes types and insulin levels is illustrated in Figure A5.


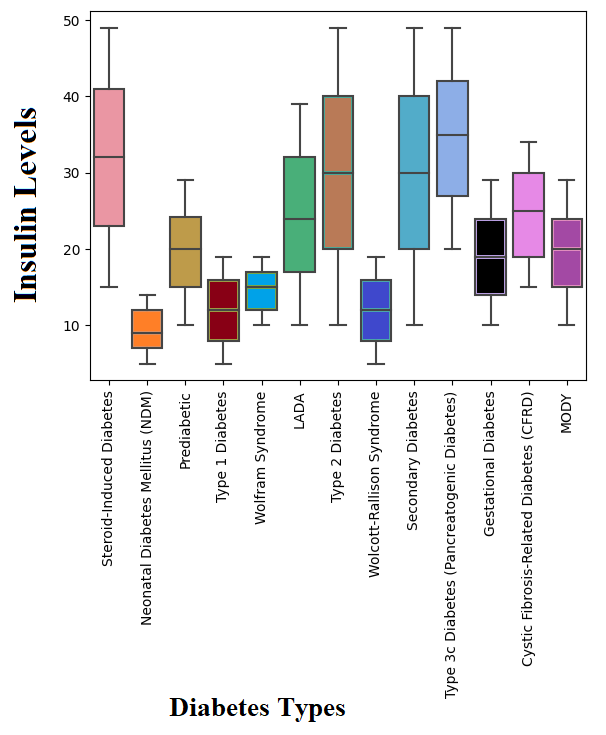


**Figure A5.** The Relationship between Diagnosis Type and Insulin Levels of diabetes_Dataset Dataset

| **Model** | **Accuracy** | **F1 Score** | **AUC** | **Training Score** | **MSE** | **R² Score** | **Observation** |
| --- | --- | --- | --- | --- | --- | --- | --- |
| DT | 89.03% | 0.8889 | 0.9812 | 1.0000 | 3.7768 | 0.7302 | Significant improvement with PSO. Interpretable and fast. |
| RF | 90.34% | 0.9023 | 0.9955 | 1.0000 | 2.6509 | 0.8106 | Strong baseline, minimal improvement post-PSO. Very reliable. |
| GB | 90.28% | 0.9068 | 0.9962 | 0.9170 | 2.5699 | 0.8164 | Best performing model overall. Excellent class-wise metrics. |
| LR | 71.40% | 0.7115 | 0.9705 | 0.7149 | 9.2702 | 0.3378 | Poor accuracy and weak fit. Minimal gains with PSO. |
| NB | 82.79% | 0.8285 | 0.9897 | 0.8256 | 5.1478 | 0.6323 | Matches RF in metrics. Fast and simple, but assumes feature independence. |

Table A3. A comparison between DT, RF, GB, LR, NB

We will present GB model as it is the best-performing model.


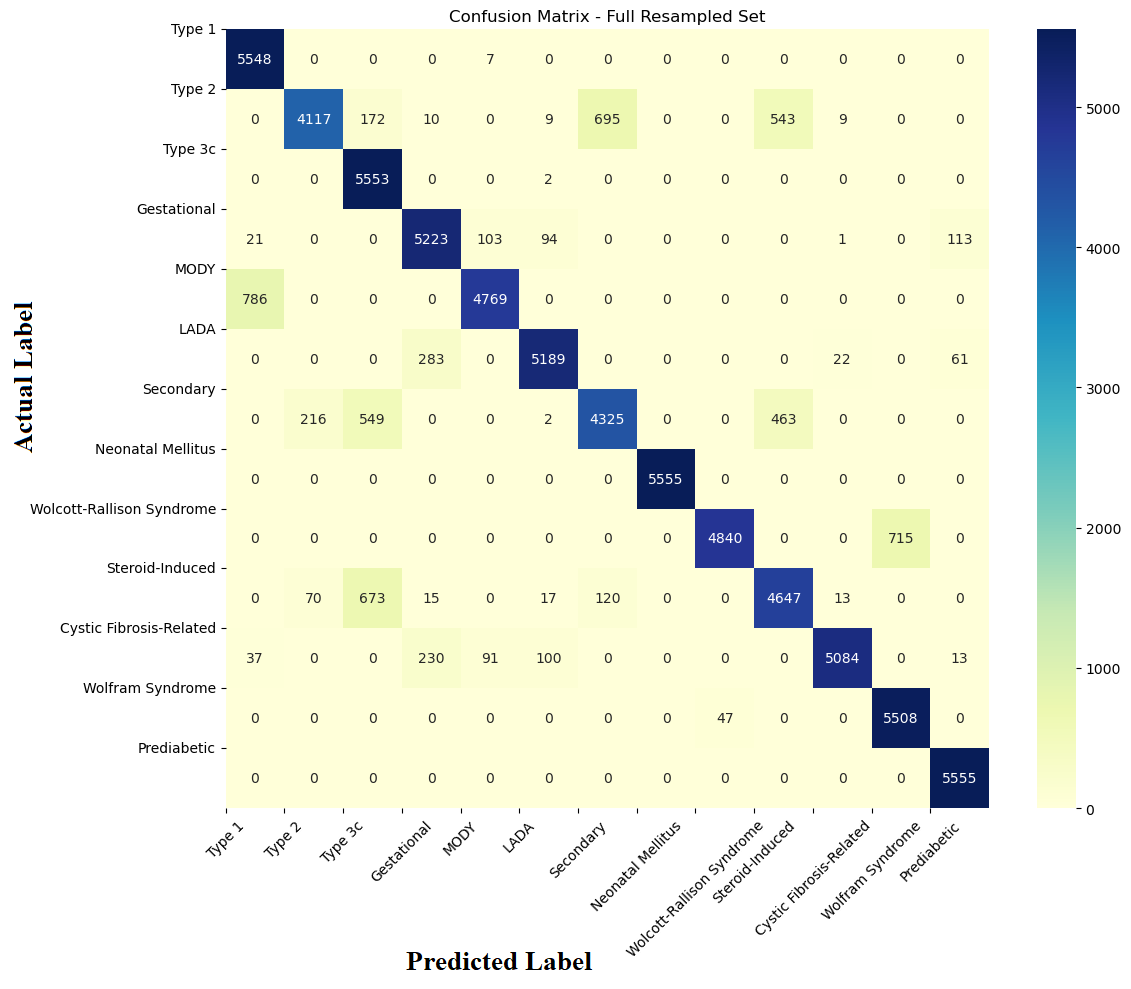


**Figure A6.** The Confusion Matrix of diabetes_Dataset Dataset

The confusion matrix for the full resampled set shows that the GB model performs well overall, with strong diagonal dominance indicating accurate predictions across most diabetes types. For instance, classes like Type 1, Type 3c, Gestational, Neonatal Mellitus, and Prediabetic exhibit very high correct classification counts (e.g., 5548, 5553, 5223, etc.) and minimal confusion with other classes. However, there is noticeable misclassification between Type 2, MODY, Steroid-Induced, and Cystic Fibrosis-Related types, with several predictions overlapping, especially with MODY and Steroid-Induced being confused for Type 2 or Type 3c, as shown in Figure A6.


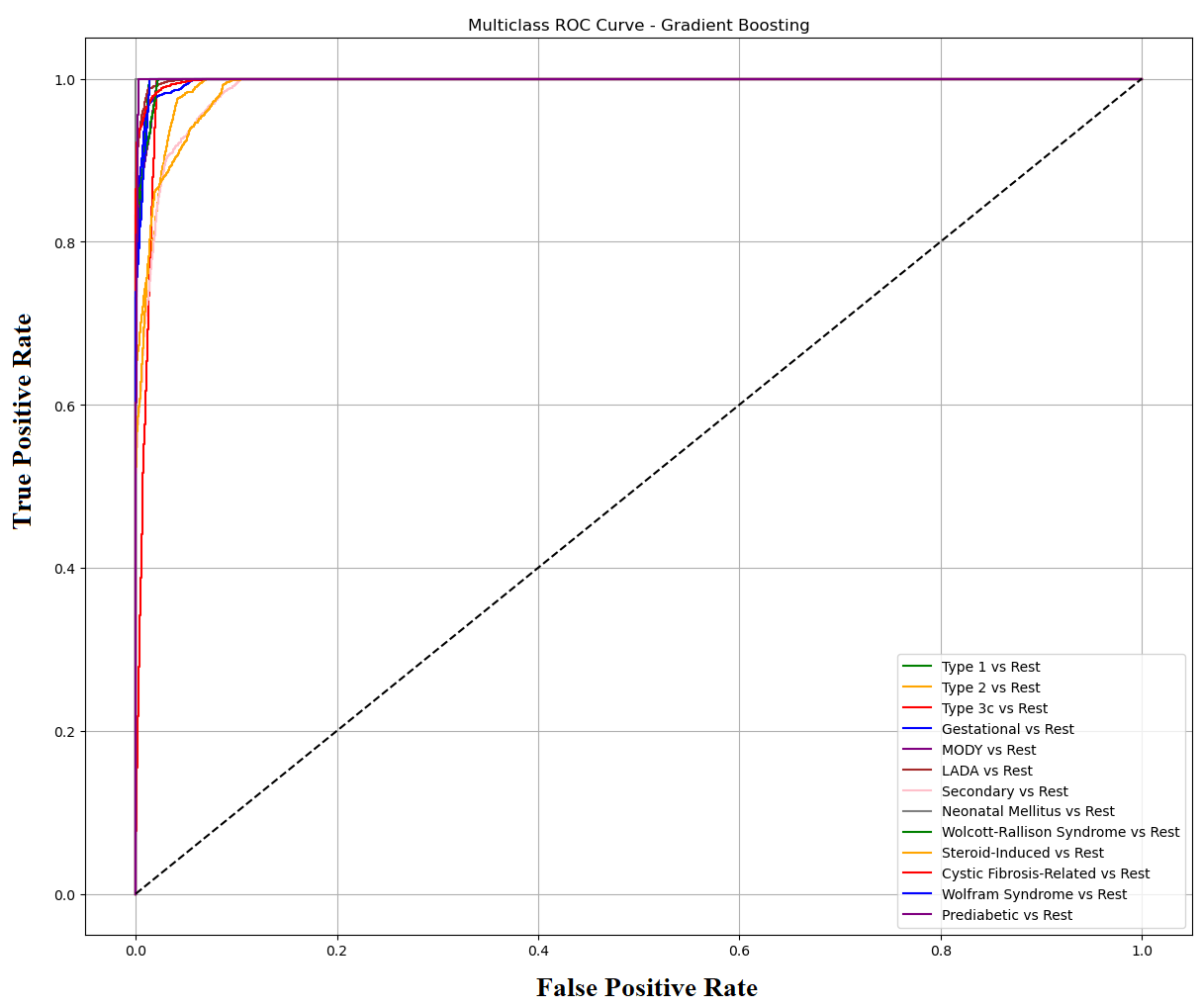


**Figure A7.** The ROC Curve of diabetes_Dataset Dataset

The multiclass ROC curve for the GB model demonstrates excellent overall classification performance across all diabetes types. Most of the curves hug the top-left corner of the plot, indicating that high TPR and very low FPR is a sign of strong discriminative power. Particularly, classes like Type 1, Gestational, Neonatal Mellitus, Wolfram Syndrome, and Prediabetic show nearly perfect separation from the rest, as their ROC curves are closest to the ideal point (0,1). However, curves for Type 2, MODY, and Steroid-Induced show slightly more deviation, implying those classes may be harder to distinguish from others. Overall, the ROC analysis supports that Gradient Boosting is a highly effective model for this multiclass classification task, as shown in Figure A7.


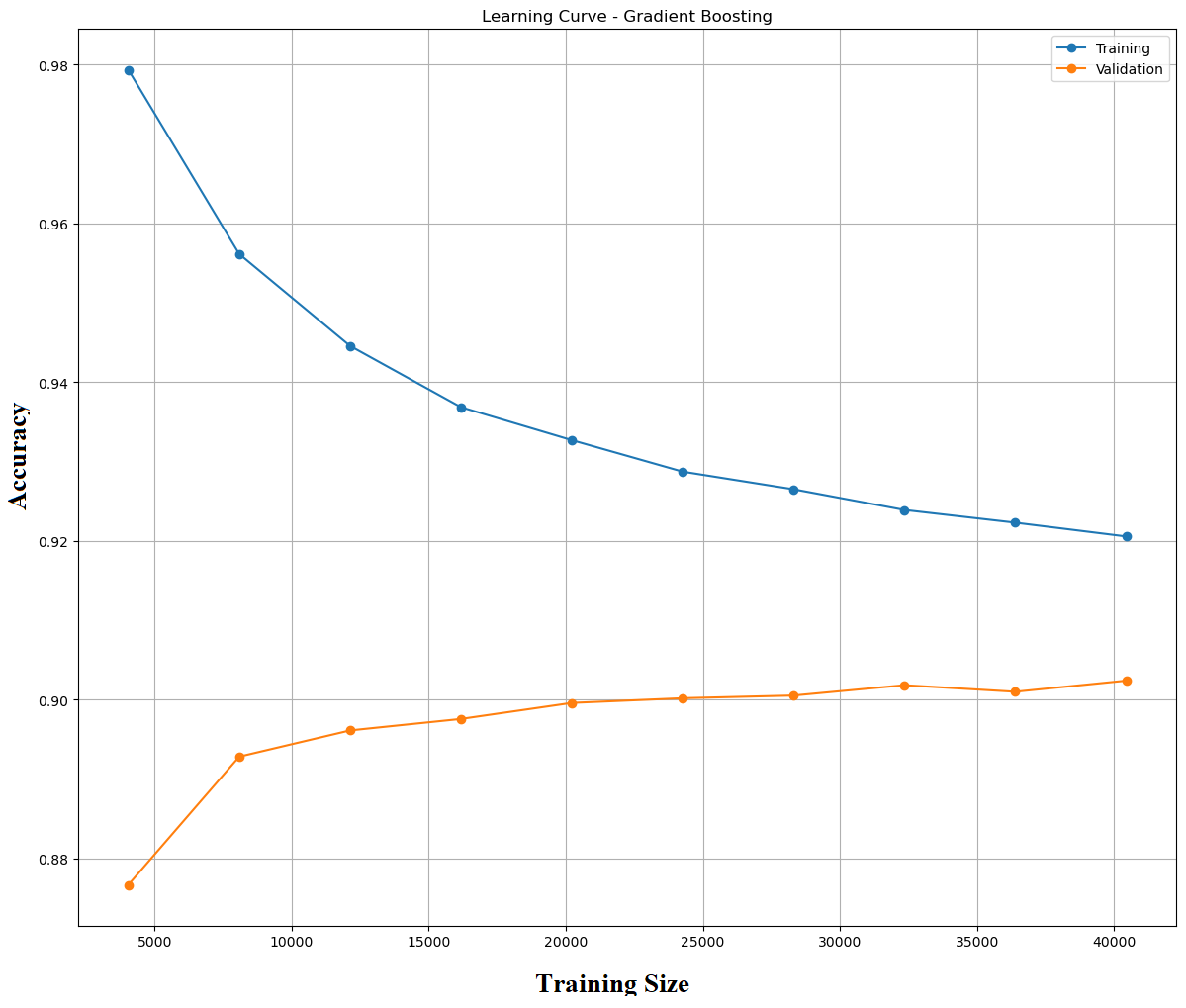


**Figure A8.** The learning Curve of diabetes_Dataset Dataset

The learning curve for the GB model illustrates a classic case of a small generalization gap with high and stable performance. As the training size increases, training accuracy slightly decreases from about 0.98 to 0.92, indicating the model is generalizing better and not overfitting to smaller datasets. Meanwhile, validation accuracy steadily improves from around 0.877 to just above 0.902 as more data becomes available, showing that the model benefits from more training samples. The convergence of training and validation curves, particularly with a narrowing gap, suggests the model is well-tuned and learning effectively, with no major signs of underfitting or overfitting. This pattern confirms that Gradient Boosting performs robustly on this multiclass classification task, as shown in Figure A8.
